# *Clostridium butyricum* population balance model: Predicting dynamic metabolic flux distributions using an objective function related to extracellular glycerol content

**DOI:** 10.1371/journal.pone.0209447

**Published:** 2018-12-20

**Authors:** Luis Miguel Serrano-Bermúdez, Andrés Fernando González Barrios, Dolly Montoya

**Affiliations:** 1 Bioprocesses and Bioprospecting Group, Universidad Nacional de Colombia, Ciudad Universitaria, Carrera, Bogotá D.C., Colombia; 2 Grupo Cundinamarca Agroambiental, Departamento de Ingeniería Ambiental, Universidad de Cundinamarca, Facatativá, Colombia; 3 Grupo de Diseño de Productos y Procesos (GDPP), Departamento de Ingeniería Química, Universidad de los Andes, Bogotá D.C., Colombia; Ecole Polytechnique, CANADA

## Abstract

**Background:**

Extensive experimentation has been conducted to increment 1,3-propanediol (PDO) production using *Clostridium butyricum* cultures in glycerol, but computational predictions are limited. Previously, we reconstructed the genome-scale metabolic (GSM) model *i*Cbu641, the first such model of a PDO-producing *Clostridium* strain, which was validated at steady state using flux balance analysis (FBA). However, the prediction ability of FBA is limited for batch and fed-batch cultures, which are the most often employed industrial processes.

**Results:**

We used the *i*Cbu641 GSM model to develop a dynamic flux balance analysis (DFBA) approach to predict the PDO production of the Colombian strain *Clostridium* sp IBUN 158B. First, we compared the predictions of the dynamic optimization approach (DOA), static optimization approach (SOA), and direct approach (DA). We found no differences between approaches, but the DOA simulation duration was nearly 5000 times that of the SOA and DA simulations. Experimental results at glycerol limitation and glycerol excess allowed for validating dynamic predictions of growth, glycerol consumption, and PDO formation. These results indicated a 4.4% error in PDO prediction and therefore validated the previously proposed objective functions. We performed two global sensitivity analyses, finding that the kinetic input parameters of glycerol uptake flux had the most significant effect on PDO predictions. The other input parameters evaluated during global sensitivity analysis were biomass composition (precursors and macromolecules), death constants, and the kinetic parameters of acetic acid secretion flux. These last input parameters, all obtained from other *Clostridium butyricum* cultures, were used to develop a population balance model (PBM). Finally, we simulated fed-batch cultures, predicting a final PDO production near to 66 g/L, almost three times the PDO predicted in the best batch culture.

**Conclusions:**

We developed and validated a dynamic approach to predict PDO production using the *i*Cbu641 GSM model and the previously proposed objective functions. This validated approach was used to propose a population model and then an increment in predictions of PDO production through fed-batch cultures. Therefore, this dynamic model could predict different scenarios, including its integration into downstream processes to predict technical-economic feasibilities and reducing the time and costs associated with experimentation.

## Background

The production of 1,3-propanediol (PDO) has been widely studied to valorize the glycerol overproduced in the biodiesel industry [1, 2]. High yield PDO production in glycerol metabolizing organisms such as *Clostridium butyricum* is limited to experimental studies involving fed-batch cultures and mutant strains obtained by random mutagenesis [3–5]. However, organism specific metabolic models can be used to rationally design strains that achieve high product yields. The genome-scale metabolic (GSM) model of a PDO-producing organism *Clostridium butyricum i*Cbu641 was recently reconstructed, containing 641 genes, 891 reactions, and 701 metabolites, and it can predict metabolic phenotypes under varying glycerol concentrations [6]. This GSM model can be used to analyze *Clostridium butyricum* metabolism and rationally design strategies to increase PDO production.

The *i*Cbu641 model predictions were validated at steady state using fermentation data, from cultures grown under different substrates, including glycerol to produce PDO. These model predictions were tested by simulating steady state conditions using flux balance analysis (FBA) and parsimonious FBA [6]. However, the steady state yield predictions do not capture the interactions between intracellular and extracellular environments nor the concentration profiles. Concentration profiles are typically predicted using conventional kinetic models; however, the complexity of the models increases as numerous kinetic parameters are needed to capture the impact of changing intracellular metabolite pools, which are difficult to measure experimentally or estimate computationally due to lack of experimental studies which explore metabolic or genetic perturbations. Millat *et*. *al*. have summarized dynamic models developed for cultures of solventogenic *Clostridium* strains and highlighted the challenges in estimation of kinetic parameters, which includes the quality and quantity of experimental data used or enzyme intracellular concentrations [7]. This led us to use dynamic flux balance analysis (DFBA) which captures mass balances at a dynamic state to analyze batch and fed-batch cultures in greater detail [8–12] as was previously shown by Mahadevan *et*. *al*. [8] using a simplified *E*. *coli* metabolic model.

*Clostridium* cultures have not been studied extensively using DFBA, where the only reported study explores butanol production using a *C*. *acetobutylicum* and *C*. *cellulolyticum* co-culture using cellulose as substrate [13]. Other products of solventogenic *Clostridium* strains, including hydrogen and PDO, have better experimental titers and yields using batch and fed-batch cultures than continuous cultures at steady state [3], but the existing models are focused on capturing predictions at steady state [14–25]. Additionally, *i*Cbu641 model predictions at steady state for cultures grown in glycerol elucidate that substrate concentration affects the PDO yields [6], which can be more effectively captured using dynamic simulation. In this study, product yields, including PDO, obtained using batch cultures have been used to test the dynamic model’s predictions.

DFBA can be solved using the dynamic optimization approach (DOA), static optimization approach (SOA), and direct approach (DA). DOA uses orthogonal collocation to solve the entire simulated culture in a single optimization, making its solution highly complex for use in relatively large GSM models, thus limiting its application [8, 26–29]. In contrast, SOA and DA divide the simulated culture into several time intervals and solve the system in each of them. As a result, these approaches are often used in GSM models like *S*. *cerevisiae* [9–11, 30–39], *E*. *coli* [8, 40–47], *S*. *cerevisiae* and *E*. *coli* cocultures [48–50], CHO cells, *Shewanella oneidensis*, *Chlamydomonas reinhardtii*, *Lactococcus lactis*, and even soil consortia [29, 51–55]. SOA integrates ordinary differential equations (ODE) using a previously predicted intracellular optimum, requiring a small step size, whereas DA solves the ODE simultaneously with intracellular behavior [56], which is an advantage in predicting fed-batch cultures [35].The above leads to suggest DA is an appropriate approach to predict PDO production over time using DFBA, which we seek to qualitatively as well as quantitatively validate based on their predictions as well as computational time requirements.

Phenotypic predictions using FBA or DFBA have some challenges in their implementation [57, 58], as with other biological models (*i*.*e*., quorum sensing and structured kinetic models). These challenges include the adequate selection of the input parameter values or the model’s capability to respond to perturbations [59]. Concerning to perturbations performed in GSM models, they are focused to increase the yield of the metabolite of interest using approaches as prediction of mutants by knockout, downregulations of genes or fed-batch cultures [32, 51, 56, 60]. However, these perturbations do not identify the significance of input parameters on DFBA predictions as do the sensitivity analyses approaches. Different sensitivity analyses approaches have been performed in other kinds of biological models to identify significance of input parameters, but the application of such approaches in DFBA predictions have been rarely considered [57, 58, 61–68]. Hence, we consider essential to perform sensitivity analyses to identify parameters with significance on DFBA predictions.

Sensitivity analyses can be either local or global. In local sensitivity analysis, the output variable model is evaluated by varying only one input parameter around a local point, while all other input parameters remain constant. However, the local approach is not recommended in biological models as we need to account for the uncertainty in all the input parameters simultaneously [57]. Global sensitivity analysis quantifies the overall effect on the model of several input parameters within large ranges, making it more appropriate for biological models [57]. Global sensitivity approaches include multi-parametric sensitivity analysis (MPSA), partial rank correlation coefficient (PRCC), the Morris method, the Sobol method, or the Fourier amplitude sensitivity test (FAST) [57, 69]. MPSA or PRCC approaches have low computational cost but are restricted to monotonic models. The Sobol method or FAST have high computational cost, and are recommended only in small-scale biological models [57]. In addition, results using low (PRCC) and high (Sobol and FAST) computational costs are highly correlated, suggesting that the results are independent of the approach selected [64]. Therefore, sensitivity analysis of DFBA predictions could be developed using approaches with low computational cost as PRCC or MPSA. MPSA uses the Kolmogorov–Smirnov (*K-S*) statistic to evaluate the significance level between each pair of output variable and input parameter, being the input parameters randomly generated by methods like Monte Carlo approach. On the other hand, PRCC calculates a positive or negative correlation between each couple of input parameter and output variable using the Pearson correlation coefficient [57]. Therefore, MPSA and PRCC results can complement each other to ensure a more robust sensitivity analysis of DFBA model.

Monte Carlo approach can be employed also to perform numerical Population balance models (PBM) besides global sensitivity analysis as MPSA or PRCC. PBM are developed to capture the heterogeneity in bioreactor caused by the cell variability in order to improve the design and control of bioprocesses [70]. From the kinetic point of view, a PBM is a segregated model where population is distributed by at least one cell characteristic as size, age, mass, etc, making segregated models highly complex [71]. Therefore, Mantzaris *et*. *al*. [72] highlight the possibility of developing this kind of segregated models by numeric randomizing of input parameters by approaches like Monte Carlo, to avoid the mathematical complexity that is characteristic of segregated models. This led us to propose a numerical PBM coupled to DBFA that predicts the heterogeneity in PDO predictions caused by cell variability.

This study describes the development of a dynamic metabolic model of *Clostridium butyricum* capable of predicting cultures to produce and accumulate PDO when grown in glycerol, comparing previously predictions using DOA, SOA, and DA approaches. A recently reconstructed GSM model (*i*Cbu641), which was validated at steady state using nonlinear objective functions [6], is used as the basis to develop the dynamic model. The predictions from the developed dynamic model were validated using fermentation data from batch cultures of the Colombian strain *Clostridium* sp IBUN 158B, isolated by our Bioprocesses and Bioprospecting Group. This strain is a natural PDO producer and has been employed over the past 20 years in several studies aimed at understanding PDO production, including proteomic analysis [73–75]. Additionally, using MPSA and PRCC approaches, the sensitivity analysis of the dynamic model revealed the key parameters which can be exploited to increase PDO production. This was complemented with the development of a PBM that quantified variability in PDO predictions. Finally, we performed perturbations in culture conditions, also in order to increase PDO production.

## Material and methods

### Bacterial strain, fermentation, and culture conditions

Experimental validation of PDO production was performed using Colombian-native strain *Clostridium* sp IBUN 158B, isolated and stored by the Bioprocesses and Bioprospecting Research Group from the Institute of Biotechnology of the Universidad Nacional de Colombia. Activation was done using sterile reinforced Clostridial medium (RCM) at pH 7 and cultured anaerobically during 12 hours at 37°C after a previous heat shock [75]. Inoculum were cultured in 100 mL vials during 24 hours at 37°C in an industrial medium with the following composition: glycerol (40 g/L), yeast extract (3 g/L), cysteine (0.5 g/L), K_2_HPO_4_ (1 g/L), KH_2_PO_4_ (0.5 g/L), biotin (4 mg/L), PABA (3 mg/L), and minerals solution (4 mL/L).

Cultures were performed in a BIOSTAT reactor with a culture volume of 1 L of industrial medium and 10% of inoculum. The following conditions were maintained constant: temperature (37°C), pH (7), agitation (90 rpm), bubbling gas (N_2_), gas flow (0.005 vvm), and dissolved oxygen (<1.5%). We performed two cultures at glycerol limitation and two cultures at glycerol excess (an initial glycerol concentration of less than and greater than 15 g/L, respectively).

### Quantification of biomass, substrate, and products

Biomass was determined indirectly by spectrophotometry at 600 nm using ThermoScientific Evolution 201, and the dry weight was calculated using the calibration curve. Substrate glycerol and the products PDO, butyric acid, acetic acid, lactic acid, and butanol were quantified using ultra-fast liquid chromatography (UFLC) with a refractive index detector (Shimadzu RID 10A) at 60°C and AMINEX HPX— 87H column (Biorad) at 63°C, a solution of 3 mM of sulfuric acid as the phase mobile, and 0.5 mL/min flow during 45 minutes of run time. The samples had a volume of 750 μL and were located in a Shimadzu Prominence LC-20AD autosampler, which ultimately injected 20 μL to UFLC. Lab Solutions software V. 1.25 (Shimadzu) was used for calculating retention time, the slope of the straight line, and linear ratio coefficients (R^2^) to determine the concentrations of the compounds evaluated.

### Dynamic predictions using DFBA

The numerical solution of DFBA requires differential equation parametrization for both the DOA and DA approaches [8, 35]. We used the Lagrange interpolating polynomial and its first derivative (Eqs 1 and 2, respectively) [76]. We evaluated the following orthogonal polynomials: Chebyshev of the first kind, Chebyshev of the second kind, Laguerre, Legendre, and Hermite. The polynomials were evaluated with six collocation points, *x*_*a*_, calculated using the *Orthopolynom* package by R Project. A higher number of collocation points would enhance the adjustment of differential equations but also increase the computational cost.

$$Y_{P}(X)=\sum_{a=1}^{P+1}\left[y_{b}(x_{a})∙l_{b}(x_{a})\right]\left\{ \begin{array}{c} y_{b}(x_{a})=\begin{array}{c} 0\;a\neq b\\ y_{b}\;a=b \end{array}\\ l_{b}(x_{a})=\prod_{\begin{array}{c} b=1\\ b\neq a \end{array}}^{P+1}\frac{x-x_{b}}{x_{a}-x_{b}} \end{array}\;\forall \;a,b\in 1,\ldots ,P+1\right.$$ Eq 1

$$\frac{d\;Y_{P}(X)}{dX}=\sum_{a=1}^{P+1}\left[y_{b}(x_{a})∙\frac{d\;l_{b}(x_{a})}{d\;x}\right]$$ Eq 2

Where: *Y*_*P*_*(X)* is the Lagrange interpolating polynomial, with Y as the output variable and X the input variable; *P+1* is the number of collocation points that pass through polynomial order *P*; the collocation point *a* is denoted by *x*_*a*_; *y*_*b*_ is the interception point *b*; and *l*_*b*_*(x*_*a*_*)* is the *b* term of the Lagrange interpolating polynomial.

Death constants (*k*_*d*_), kinetic adjustments of the upper bound of acetic acid secretion flux (Eq 3), and glycerol uptake flux (Eq 4) were used as constraints of the dynamic model. The summary of kinetic parameters is shown in Table 1. Death constants were calculated using data from Solomon *et al*. [77], whereas acetic acid constraint was previously adjusted [6] using experimental data from Solomon *et al*. [77] and Papanikolaou *et al* [78]. Regarding the glycerol kinetic model, this was adjusted using only glycerol profiles of three cultures: the first was one culture at glycerol limitation, the second was one culture at glycerol excess, both from this study, the third culture was performed at glycerol excess by Aragon [79]. Additionally, we used the general Eq 5 to describe both objective functions *Z* used, where *w* depends on glycerol concentration: thus the first objective function is at glycerol limitation (*w* = 1) and the second objective function is at glycerol excess (*w* = 0.04) [6]. The DFBA was solved using the DOA, SOA, and DA approaches, as shown in Eqs 6, 7 and 8, respectively [8, 35]. Despite the objective functions employed are non-convex, Schuetz *et al*. suggested that the predicted local optimum is indeed the global optimum [80], which also we validated previously [6]. Regarding the constraints, Eqs 3 and 4 are the only nonlinear constraints employed in dynamic models, where Eq 3 is a logistic model and Eq 4 is a Ghose and Tyagi model, which are both convex functions, meaning they do not affect the global optimum calculated by the three approaches evaluated.

**Table 1:** Summary of kinetic parameters used in the DFBA model with glycerol as the only carbon source.

Parameter	Description	Value	Units	Origin of data
$v_{0}^{aa}$	Basal flux of acetic acid secretion (glycerol uptake flux trends to zero)	0.1578	mmol/g·h	[77, 78]
$v_{\infty }^{aa}$	Maximum flux of acetic acid secretion (glycerol uptake flux trends to infinite)	11.50	mmol/g·h	[77, 78]
*R*^*aa*^	Accumulation rate of acetic acid secretion flux in function of glycerol uptake flux	0.0859	g·h/mmol	[77, 78]
$k_{d}^{lim}$	Death constant at glycerol limitation	0.0350	h^-1^	[77]
$k_{d}^{exc}$	Death constant at glycerol excess	0.0105	h^-1^	[77]
$v_{max}^{Gly}$	Maximum flux of glycerol uptake	174.86	mmol/g h	This study, [79]
$k_{s}^{Gly}$	Affinity constant of glycerol uptake flux to glycerol concentration	482.1	mM	This study, [79]
$k_{I}^{Gly}$	Inhibition constant of glycerol uptake flux to glycerol concentration	755.4	mM	This study, [79]

$$v_{A.Ac.}^{max}=\frac{v_{\infty }^{aa}∙v_{0}^{aa}∙e^{\left(R^{aa}∙v_{Gly}\right)}}{v_{\infty }^{aa}+v_{0}^{aa}∙[e^{\left(R^{aa}∙v_{Gly}\right)}-1]}$$ Eq 3

$$v_{Gly}=\left(\frac{v_{max}^{Gly}∙[Glycerol]}{k_{s}^{Gly}+[Glycerol]}\right)∙\left(1-\frac{\left[Glycerol\right]}{k_{I}^{Gly}}\right)$$ Eq 4

$$Z=\frac{\mu }{\left(w∙\sum_{j=1}^{N}v_{j}^{2}+(1-w)∙v_{ATP\;prod}^{2}\right)}$$ Eq 5

$$\begin{array}{l} Max\sum_{g=0}^{G}\int_{0}^{t_{f}}\left[Z∙\delta (t-t_{g})\right]\;dt\\ Subject\;to\\ \;\left\{ \begin{array}{l} \frac{dz_{i}}{dt}=-\sum_{j=1}^{N_{Exchange}}S_{ij}∙v_{j}∙X\;\forall \;i\in 1,\ldots ,M_{Extracellular}\\ \frac{dX}{dt}=(\mu -k_{d})∙X\\ \begin{array}{l} \sum_{j=1}^{N}S_{ij}∙v_{j}=0\;\forall \;i\in 1,\ldots ,M\\ \mu =\sum_{j=1}^{N}c_{j}∙v_{j}\\ \begin{array}{l} v_{j}^{min}<v_{j}<v_{j}^{max}\;\forall \;j\in 1,\ldots ,N\\ \hat{c}(v,z)\leq 0\\ \begin{array}{l} z_{i}(t)\geq 0\;z_{i}(t_{0})=z_{i,0}\;\forall \;i\in 1,\ldots ,M_{Extracellular}\\ X(t)\geq 0\;X(t_{0})=X_{0}\\ t_{g}=t_{0}+g∙\frac{t_{f}-t_{0}}{G}\;\forall \;g\in 0\ldots .G \end{array} \end{array} \end{array} \end{array}\right. \end{array}$$ Eq 6

Where: *M* is the total number of metabolites (*M*_*extracellular*_ are the extracellular metabolites); *N* is the total number of reactions (*N*_*exchange*_ are the exchange reactions); *S*_*ij*_ is the stoichiometric coefficient of metabolite *i* in reaction *j*; *v*_*j*_ is the flux value in which this reaction occurs; *v*_*j*_^*max*^ and *v*_*j*_^*min*^ are the upper and lower bounds of the flux *v*_*j*_; *z*_*i*_ is the extracellular concentration of metabolite *i* (*z*_*i*,*0*_ is the initial concentration); X is the biomass concentration (*X*_*0*_ is the initial concentration); *μ* is the specific growth rate; *c*_*j*_ is the weight of reaction *i* in growth rate; *ĉ(v*,*z)* is the vector of nonlinear constraints, which in this case are Eqs 3 and 4; the initial and final times of simulated culture are *t*_*0*_ and *t*_*f*_. *δ(t—t*_*g*_*)* is the Dirac delta function; and *G* is the total number of intervals in which the culture time was discretized.

$$\begin{array}{l} Max\;Z\;\forall \;t_{g}\in [t_{0},t_{f}]\\ Subject\;to\\ \;\left\{ \begin{array}{l} z_{i}(t+∆t)=z_{i}(t)-\sum_{j=1}^{N_{Exchange}}S_{ij}∙v_{j}(t)∙X(t)∙∆t\\ \;\forall \;i\in 1,\ldots ,M_{Extracellular}\\ \begin{array}{l} X(t+∆t)=X(t)+(\mu -k_{d})∙X(t)∙∆t\\ \begin{array}{l} \sum_{j=1}^{N}S_{ij}∙v_{j}=0\;\forall \;i\in 1,\ldots ,M\\ \mu =\sum_{j=1}^{N}c_{j}∙v_{j} \end{array}\\ \begin{array}{l} v_{j}^{min}<v_{j}<v_{j}^{max}\;\forall \;j\in 1,\ldots ,N\\ \hat{c}(z_{i}(t),v_{j}(t))\leq 0\\ \begin{array}{l} z_{i}(t)\geq 0\;z_{i}(t_{0})=z_{i,0}\;\forall \;i\in 1,\ldots ,M_{Extracellular}\\ X(t)\geq 0\;X(t_{0})=X_{0}\\ ∆t=\frac{t_{f}-t_{0}}{G}\;\forall \;g\in 0\ldots .G \end{array} \end{array} \end{array} \end{array}\right. \end{array}$$ Eq 7

$$\begin{array}{l} Max\;Z\;\forall \;t_{g}\in [t_{0},t_{f}]\\ Subject\;to\\ \;\left\{ \begin{array}{l} \frac{dz_{i}}{dt}=-\sum_{j=1}^{N_{Exchange}}S_{ij}∙v_{j}∙X\;\forall \;i\in 1,\ldots ,M_{Extracellular}\\ \frac{dX}{dt}=(\mu -k_{d})∙X\\ \begin{array}{l} \sum_{j=1}^{N}S_{ij}∙v_{j}=0\;\forall \;i\in 1,\ldots ,M\\ \mu =\sum_{j=1}^{N}c_{j}∙v_{j}\\ \begin{array}{l} v_{j}^{min}<v_{j}<v_{j}^{max}\;\forall \;j\in 1,\ldots ,N\\ \hat{c}(v,z)\leq 0\\ \begin{array}{l} z_{i}(t)\geq 0\;z_{i}(t_{0})=z_{i,0}\;\forall \;i\in 1,\ldots ,M_{Extracellular}\\ X(t)\geq 0\;X(t_{0})=X_{0}\\ t_{g}=t_{0}+g∙\frac{t_{f}-t_{0}}{G}\;\forall \;g\in 0\ldots .G \end{array} \end{array} \end{array} \end{array}\right. \end{array}$$ Eq 8

### Global sensitivity analysis development

We evaluated the composition of 44 biomass precursors (seven fatty acids, 20 amino acids, eight nucleotides, three polar lipids, and six cofactors) and eight macromolecules (proteins, DNA, RNA, lipids, teichoic acid, peptidoglycans, carbohydrates, and pool of traces) expressed by the variation of their stoichiometric coefficients in the GSM model. We included the eight kinetic parameters shown in Table 1, for a total of 60 input parameters. The DFBA model was performed using *K* different combinations of input parameter values randomly generated using normal distributions with a relative standard deviation (RSD) of 30%, excepting the kinetic parameters of glycerol uptake flux, which had a RSD of 20%. In total, the global sensitivity analyses used 2280 predicted profiles.

The first sensitivity analysis developed was MPSA, as proposed by Zi *et*. *al*. [68], who used the *K-S* statistic. Initially, we calculated for each profile *k* the mean squared error (MSE) (Eq 9), which compares the experimental *x*_*exp*_ and predicted *x*_*pred*_ values. The experimental values are from the first culture at glycerol excess (6 data). The *K* values of MSE were compared with a threshold value, which was calculated by error propagation (*i*.*e*., (6 g/L)^2^, (0.057 g/L·h)^2^, and (0.052 mol/mol)^2^ for PDO production, PDO productivity (Q_PDO_), and glycerol conversion to PDO (Y_PDO/S_), respectively). The *k* profile was classified as “unacceptable” if its MSE value was greater than the threshold; conversely, it was “acceptable” if the MSE value was less than the threshold. Then, the maximum distance between cumulative frequency distributions of “acceptable” and “unacceptable” cases was calculated as the *K-S* value for each pair of input parameter and output variable. A high *K-S* value for a given input parameter indicates a high sensitivity of the output variable to that parameter [68]. We calculated the critical value of the *K-S* statistic (*D*_*K-S*_), which depends on the number *K* of profiles, as shown in Eq 10. If the *K-S* value of an input parameter is less than the *D*_*K-S*_, its “acceptable” and “unacceptable” distributions are statistically equal.

$$SEC(k)=\sum_{i=1}^{n}{\left(x_{exp}(i)-x_{pred}(i,k)\right)}^{2}\;\forall \;k\in 1,\ldots ,K$$ Eq 9

$$D_{K-S}=\frac{1.36}{\sqrt{K}}$$ Eq 10

We performed PRCC sensitivity analysis and calculated the Pearson correlation coefficient, which is the relation between the covariance and variances of each pair of the input parameter and output variable. The PRCC sensitivity analysis evaluated only the output variables Q_PDO_ and Y_PDO/S_. The PDO profile was excluded, since the PRCC analysis is static.

### Development of a segregated approach for the dynamic model

We created random profiles with the Monte Carlo approach using an RSD of 30%, similar to the global sensitivity analysis. We produced four groups of profiles. In the first group, we perturbed only the 44 precursors’ input parameters, while the other input parameters were maintained as constants. In the other groups, the eight macromolecules, the two death constants, and the three kinetic parameters of acetic acid secretion were perturbed similarly. These 57 input parameters were considered in the development of the PBM [70]; the three kinetic parameters of glycerol uptake flux were excluded.

We also perturbed the input parameters of precursors and macromolecules, varying the RSD to 10, 20, and 30% in order to evaluate the biomass composition effect on PDO formation and biomass molecular weight. Finally, we compared the experimental values of PDO production with the PBM profiles in which we varied biomass composition (precursors and macromolecules content), age (death constants), and capability to produce acetic acid. Therefore, the dynamic prediction model was both structured and segregated.

### Perturbation in culture conditions

We performed a complete factorial design to predict a reduction in fermentation time in batch cultures, evaluating 15 initial concentrations of inoculum and 13 initial concentrations of glycerol. We simulated fed-batch cultures to predict an increment in the final PDO concentrations. The objective function *Z* was maintained; however, some constraints were modified (Eq 11) due to the dependence on time of both the reactor volume *V* and feeding flow *F*.

$$\begin{array}{l} Max\;Z\;\forall \;t_{g}\in \left[t_{0},t_{f}\right]\\ Subject\;to\\ \;\left\{ \begin{array}{l} \frac{d\left(V∙z_{i}\right)}{dt}=F∙S_{F,i}-\sum_{j=1}^{N_{Exchange}}V∙S_{ij}∙v_{j}∙X\\ \;\forall \;i\in 1,\ldots ,M_{Extracellular}\\ \frac{d\left(V∙X\right)}{dt}=V∙\left(\mu -k_{d}\right)∙X\\ \frac{dV}{dt}=F\\ F=\{\begin{array}{l} \alpha \;Constant\;feeding\;rate\\ \beta ∙v_{H^{+}}∙X∙V\;Feeding\;rate\;coupled\;to\;pH\;control \end{array}\\ \sum_{j=1}^{N}S_{ij}∙v_{j}=0\;\forall \;i\in 1,\ldots ,M\\ \mu =\sum_{j=1}^{N}c_{j}∙v_{j}\\ v_{j}^{min}<v_{j}<v_{j}^{max}\;\forall \;j\in 1,\ldots ,N\\ \hat{c}\left(v,z\right)\leq 0\\ z_{i}\left(t\right)\geq 0\;z_{i}\left(t_{0}\right)=z_{i,0}\;\forall \;i\in 1,\ldots ,M_{Extracellular}\\ X\left(t\right)\geq 0\;X\left(t_{0}\right)=X_{0}\\ V\left(t\right)\leq V_{max}\;V\left(t_{0}\right)=V_{0}\\ t_{g}=t_{0}+g∙\frac{t_{f}-t_{0}}{G}\;\forall \;g\in 0\ldots .G \end{array}\right. \end{array}$$ Eq 11

Where: *F* is the feeding flow (expressed in L/h), *α* and *β* are the constants of proportionality at constant feeding flow and feeding flow coupled to pH control, respectively; *v*_*H+*_ is the proton secretion flux; *V* is the reactor volume in the instant *t* (*V*_*0*_ is the initial volume and *V*_*max*_ is the maximum capacity of the reactor); and *S*_*F*,*i*_ is the concentration of metabolite *i* in the feeding flow *F*.

As is observed in Eq 11, we evaluated two feeding flow strategies. The first considered a constant flow *α* during the entire culture. We performed a complete factorial design where we evaluated the glycerol mass percentage in the feeding flow in addition to the *α* value. We assumed the initial and the maximum volume were 1 L and 1.5 L, respectively, and we avoided growth limitation by nitrogen or phosphorus starvation by adding these substrates in the feeding flow. The second strategy was feeding flow coupled to pH control. The other constraints used during constant feeding flow were maintained the same. This complete factorial design evaluated the mass content of glycerol in the feeding flow and the proportionality factor *β*.

### Technical implementation

DFBA was computer simulated using GAMS (General Algebraic Modeling System, GAMS Development Corp., Washington, DC) software V.24.2.2 r44857 for Linux with solver CONOPT v3.15N. Data were analyzed using Microsoft Excel 2010.

## Results and discussion

### Development and validation of a dynamic model using DFBA

**Fig 1:**
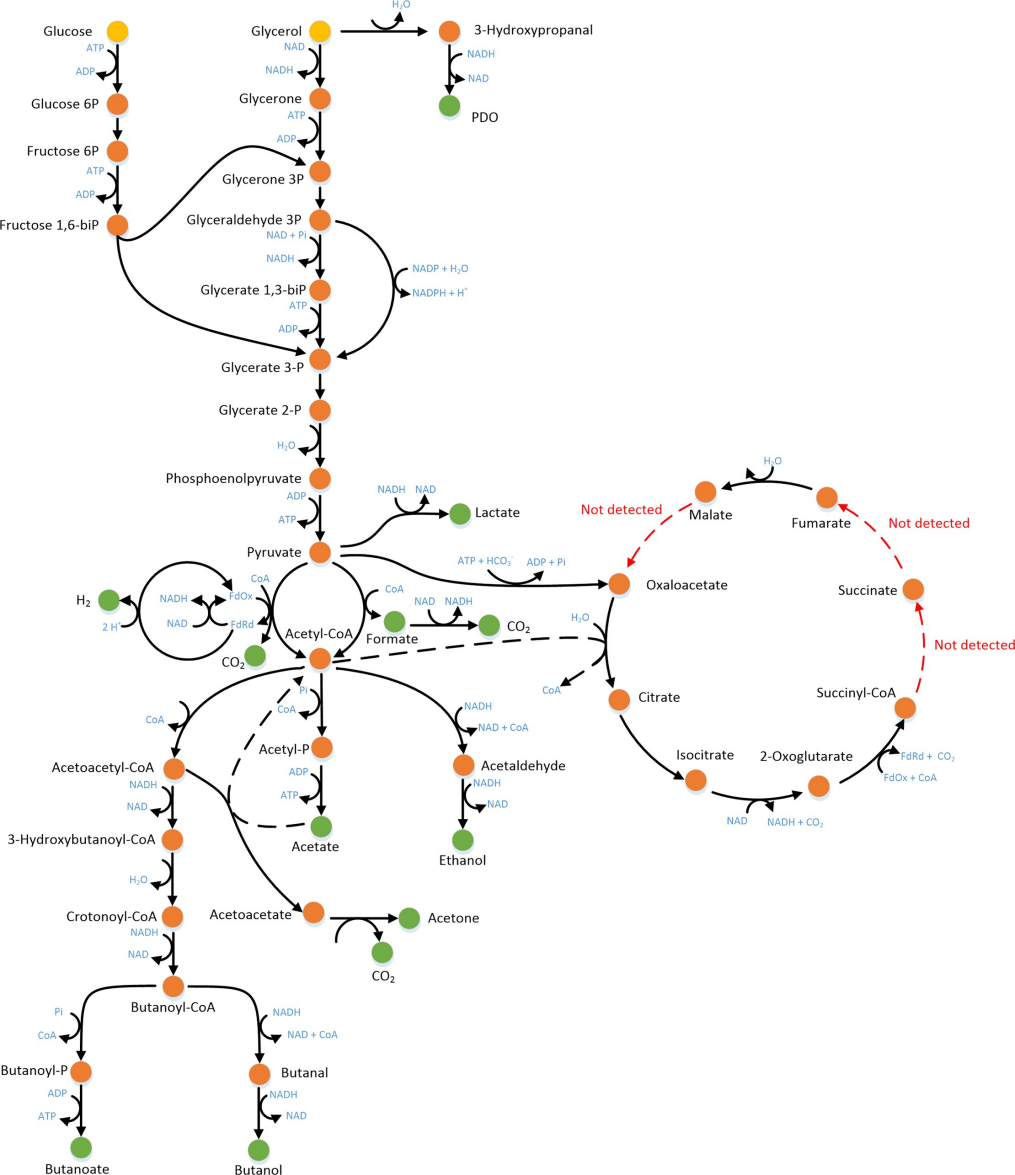
Anaerobic metabolism of glycerol by *Clostridium butyricum* of the iCbu641 GSM model. Notation: substrates (yellow dots), intracellular metabolites (orange dots), extracellular products (green dots) [6].

**Fig 2:**
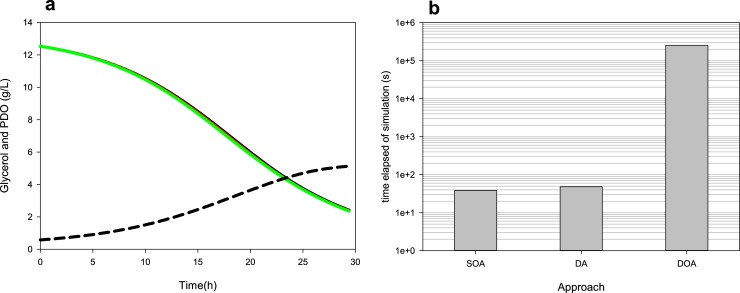
Comparison DFBA predictions using the DOA, SOA, and DA approaches. **(a)** Glycerol consumption (continuous lines) and PDO production (dashed lines) profiles using DOA (green lines), SOA (black lines), and DA (red lines) approaches. **(b)** Elapsed time during DFBA solution using different approaches.

The *i*Cbu641 GSM model [6] and DFBA were employed to predict PDO production over time. The central metabolism of *i*Cbu641 is shown in Fig 1. The orthogonal collocation method was solved using the Legendre polynomial; other polynomials were evaluated but did not yield any differences (See Fig A in S1 File for complete profiles). Predictions of a glycerol limitation culture obtained using SOA, DOA, and DA solutions appear in Fig 2A. Time profiles using the three approaches have a maximum difference of 0.07 g/L in the final PDO predicted. However, the DOA solution was the slowest, requiring approximately 5200-fold the time used by SOA and DA solutions, as shown in Fig 2B. Additionally, as we described previously [6], two objective functions are needed to predict PDO production during some scenarios of glycerol consumption, this is opposite to DOA requirements, which uses only one objective function over the entire simulated culture time. Therefore DOA cannot be employed in scenarios with change of objective function due to substrate consumption. On the other hand, despite SOA is the simplest approach, the reduction of the time required was not significant. Therefore, the DA solution was selected, which additionally can capture model perturbations, as shown with fed-batch cultures [8, 35].

**Fig 3:**
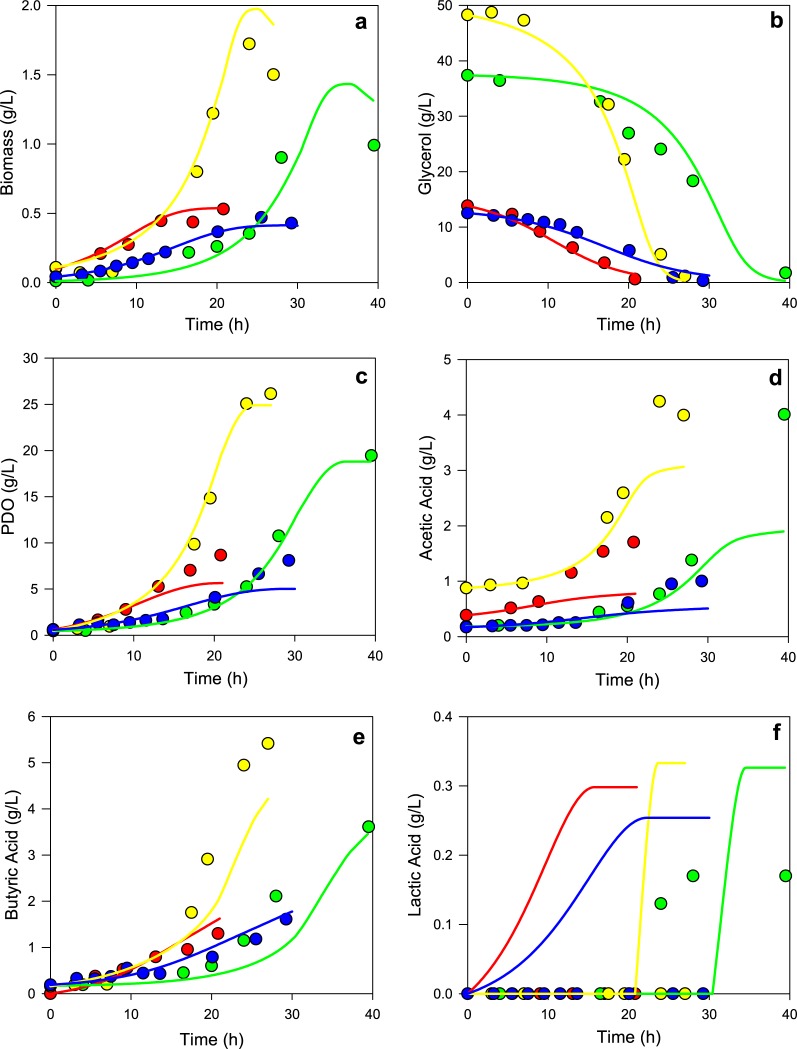
Comparison of DFBA predictions and experimental profiles of *Clostridium* sp IBUN 158B cultured at different glycerol conditions. **(a)** Biomass formation. **(b)** Glycerol consumption. **(c)** PDO formation. **(d)** Acetic acid formation. **(e)** Butyric acid formation. **(f)** Lactic acid formation. Notation: culture 1 at glycerol limitation (blue lines), culture 2 at glycerol limitation (red lines), culture 1 at glycerol excess (green lines), and culture 2 at glycerol excess (yellow lines).

Fig 3 shows dynamic predictions and experimental data of four different *Clostridium* sp IBUN 158B batch cultures, the first two at glycerol limitation and the other two at glycerol excess. In general, the predictions of glycerol consumption and biomass, PDO, and butyric acid production are similar to experimental values, validating the results obtained at steady state, where objective functions were proposed [6]. Nevertheless, for both cultures at glycerol limitation, an underestimation in glycerol consumption and PDO formation was observed when the glycerol concentration was less than 5 g/L. This underestimation is caused by the incorrect parameters estimation of the kinetic model of glycerol uptake flux from experimental data, since at low glycerol concentrations (*i*.*e*., from 0 to 5 g/L) the adjustment error was higher than the average adjustment error (See Fig A in S2 File for kinetic adjustment). This adjustment error could be caused due to constraints such as feedback inhibition or activation were not included in the kinetic model. There is also the non-growth-associated maintenance (NGAM) flux effect becoming significant on metabolic flux distribution prediction when substrate uptake flux is small [81]. This is because a high percentage of substrate consumption is directed to maintenance, and a lower fraction is directed to cellular growth and therefore to the formation of products such as PDO. This yield variation caused by the constraints is in agreement with results of Klamt *et al*. [82], who mathematically validated that fluxes and yields can act differently because of constraints, such as NGAM, and therefore proposed a linear-fractional programming (LFP) approach to maximize a yield instead a flux.

**Fig 4:**
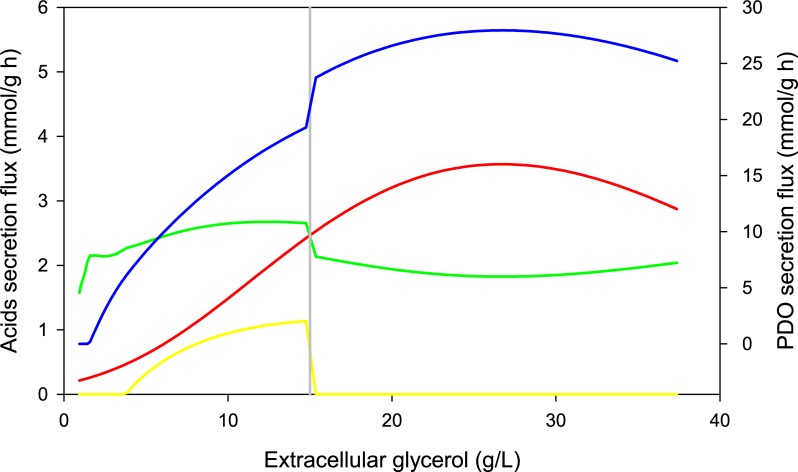
Comparison of some predicted fluxes by DFBA in the function of extracellular concentration of glycerol. Notation: PDO secretion flux (blue line), acetic acid secretion flux (red line), butyric acid secretion flux (green line), and lactic acid (yellow line). Vertical line denotes the change from suboptimum to optimum phenotype when glycerol falls to 15 g/L.

Fig 3D shows significant differences between the predicted and experimental values of acetic acid starting from the decelerated growth phase (*i*.*e*. at small glycerol uptake flux). This is due to the allosteric model employed as the upper bound of acetic acid secretion flux in the function of glycerol uptake flux (See Fig B in S2 File for kinetic adjustment). The last can be interpreted as error propagation from glycerol uptake flux kinetics to allosteric kinetics at low glycerol concentration levels. This allosteric constraint was previously adjusted from steady-state cultures in order to capture the mechanism used by *Clostridium butyricum* to control acetyl-CoA/CoA and ATP/ADP ratios, allowing to predict butyric acid secretion [6, 83]. The allosteric trend of acetic acid secretion flux is presented in Fig 4, which also shows the secretion flux of butyric acid, lactic acid and PDO as a function of glycerol concentration. The phenotypic change observed when glycerol falls to 15 g/L is described previously when glycerol is in limitation or in excess [77], and it corresponds to objective functions change we previously proposed [6]. From Fig 4, it is observed that PDO production is favored at glycerol excess conditions (>15 g/L), along with a higher acetic acid secretion flux than butyric acid secretion flux. This observation is consistent with studies of enzymatic activities, which indicate a slow decrease in activity of butyric acid forming thiolase (EC.2.3.1.9), but an increase in activity of acetate kinase (EC.2.7.2.1), PDO dehydrogenase (EC.1.1.1.202) and glycerol dehydratase (EC.4.2.1.30) with increasing glycerol uptake flux [84, 85].

Finally, the dynamic model predicted lactic acid formation at glycerol limitation conditions, as shown in Fig 3F. This is caused by the allosteric constraint of acetic acid secretion flux, which forces the prediction of proton secretion through other acids as lactic acid, since acetic acid secretion is reduced at low levels of glycerol concentrations, as is also observed in Fig 4. However, the maximum concentration predicted of lactic acid was 0.3 g/L, which means this value could fall to 0 due to error propagation described previously, making negligible the lactic acid predicted during *Clostridium* sp IBUN 158B cultured in glycerol. Although lactic acid formation implies NADH consumption and competes with PDO formation, this favors biomass formation [83, 86]. Conversely, lactic acid was detected experimentally at the end of only one culture (the longest) at glycerol excess, implying a possible stress during this culture in particular. The experimental value was 0.17 g/L, suggesting that lactic acid does not compete substantially during PDO formation by the *Clostridium* sp IBUN 158B strain, which is in agreement with dynamic predictions. Regarding the possible stress described above, we can suggest it was caused by a reduction of the anaerobiosis during this culture after the 20^th^ hour. The hypothesis is supported by observations of Chatzifragkou *et*. *al*. [87], who found that an inefficient mechanism of anaerobiosis during culture increments the lactate production.

### Global sensitivity analysis and population balance model development

**Fig 5:**
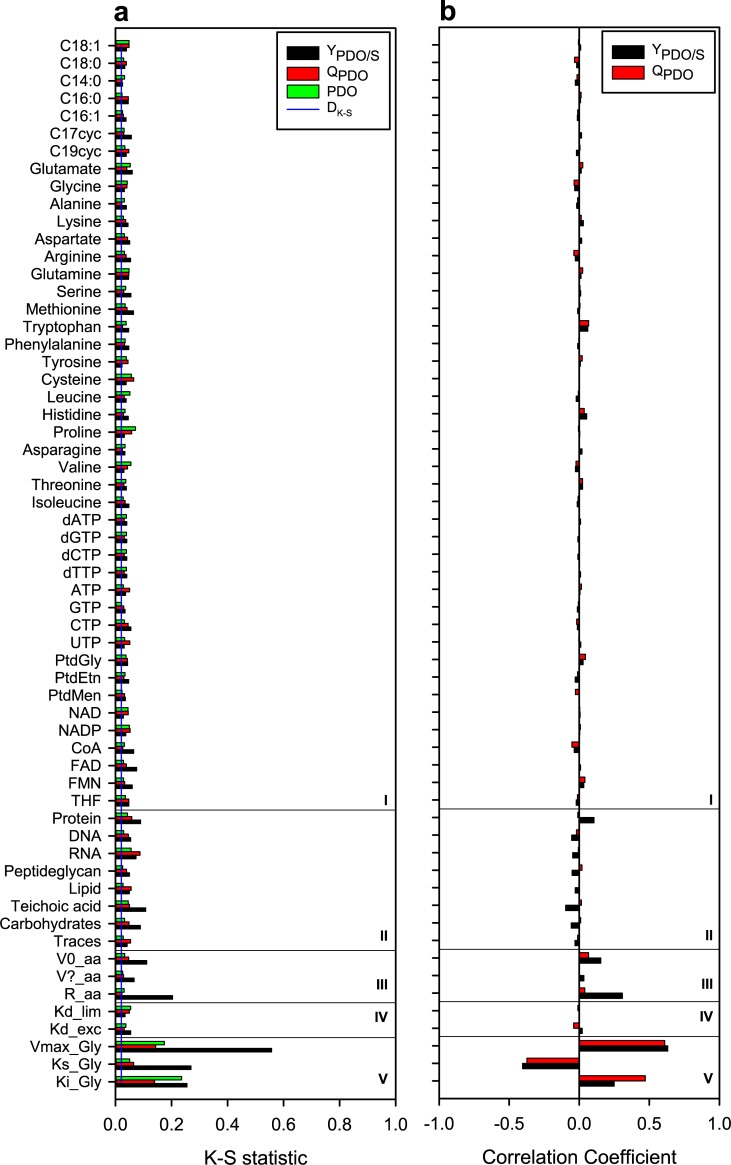
Results of global sensitivity analyses of the dynamic model. **(a)** MPSA global sensitivity analysis. **(b)** PRCC global sensitivity analysis. The input parameters were grouped as follows: 44 precursors (I), eight macromolecules (II), three kinetic parameters of acetic acid secretion flux (III), two kinetic parameters of cellular death (IV), and three kinetic parameters of glycerol uptake flux (V).

We performed global sensitivity analysis on all 60 input parameters obtained from experimental information due to the uncertainty around such parameters [57, 58]. Three input parameters related to glycerol uptake flux were calculated from *Clostridium* sp IBUN 158B cultures in glycerol. The other 57 input parameters evaluated are: two death constants (the first one for glycerol excess and the second one for glycerol limitation), kinetic parameters associated with acetic acid secretion (three input parameters), and stoichiometric coefficients of precursors in biomass formation (44 precursors and 8 macromolecules). These 57 input parameters were obtained from other *Clostridium* cultures [22, 77, 78]. We obtained 2280 PDO production profiles via simultaneous perturbation of the 60 input parameters described above (See Fig A in S3 File for complete profiles). MPSA was the first global sensitivity analysis; the Kolmogorov–Smirnov (*K-S*) statistic results of different output variables are shown in Fig 5A. PRCC was the second global sensitivity analysis; Fig 5B presents the Pearson coefficient correlation results. The MPSA and PRCC results are correlated, therefore any of these can the employed in sensitivity analysis of GSM models. However their results complement each other: MPSA quantifies which input parameters are significant and which are not, meanwhile PRCC determines the positive or negative effect of each input parameter.

Fig 5A shows that all input parameters have effect on output variables, since K-S values are higher than critical value (D_K-S_), which is presented as a blue line. Fig 5 also shows that glycerol uptake kinetic parameters are the most significant in PDO production and culture time predictions. The maximum glycerol uptake flux (*V*_*max*_^*Gly*^) and the inhibition constant (*K*_*i*_^*Gly*^) were positively correlated to PDO production, while the glycerol affinity constant was negatively correlated (*K*_*s*_^*Gly*^) to PDO production, which is consistent to other previously reported metabolic models [9, 88]. The effects of precursors, macromolecules, and death constants on output variables were lower and similar to results at steady state [6] and results reported by Hjersted and Henson [38], who evaluated two different biomass compositions and obtained a difference of 1.8% between biomass profiles. Finally, the accumulation rate (*R*^*aa*^) and initial value (*V*_*0*_^*aa*^) of acetic acid secretion flux had a positive effect on glycerol conversion to PDO (Y_PDO/S_), indicating correlation between PDO and acetic acid fluxes, as shown in Fig 4, and as reported by Zeng [89].

Concisely, input parameters with the highest significance were calculated from *Clostridium* sp IBUN 158B cultures in glycerol. Conversely, the remaining 57 input parameters, which were obtained from other *Clostridium* cultures, had the lowest effect on output variables. In other words, this dynamic model is robust because of the low impact of perturbing these 57 input parameters. Otherwise, they could not be considered in the dynamic model and we would have to calculate them specifically for *Clostridium* sp IBUN 158B cultured in glycerol, which would be experimentally demanding.

**Fig 6:**
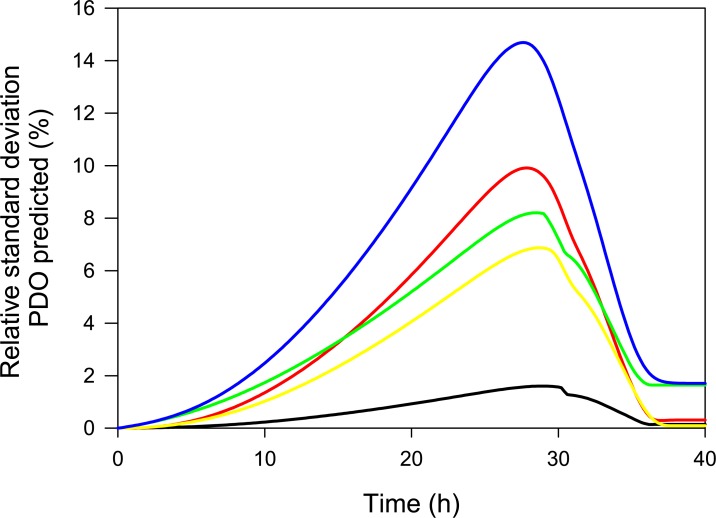
Profiles of relative standard deviations (RSDs) of predicted PDO formation for perturbation in input parameters. Notation: 44 precursors (black line), eight macromolecules (red line), two death constants (yellow line), three kinetic parameters of acetic acid secretion flux (green), 57 input parameters simultaneously (blue line). RSD for all input parameters was 30%.

Subsequently, the 57 input parameters obtained from other *Clostridium* cultures were analyzed separately and used in combination to develop a population balance model (PBM) (See S4 File). However, one of the main challenges developing a PBM is the selection of the number of cells to model so as to avoid affecting the overall prediction [90]. Danø *et*. *al*. [91] reported that 1000 cells were sufficient. This agrees with the results we obtained, where PBM predictions varied less than 0.35% between 900 and the maximum number of modeled cells (3300) (See Fig A in S4 File). Regarding the PBM results, Fig 6 shows the relative standard deviation (RSD) of PDO production in the function of culture time obtained from profiles shown in Fig B in S4 File. Results are consistent with global sensitivity analysis, perturbation in the precursor composition (black line) caused the smallest variation in the PDO formation profile. Perturbation in the macromolecules’ composition (red line) and death constants (yellow line) both have a higher effect on the PDO profile; however, predictions anticipate that their effect on final PDO concentration is up to 0.3%, similar to the steady-state results [6]. Fig 6 also shows that the maximum RSD is predicted at decelerated growth phase. Finally, according to PBM predictions the acetic acid kinetic constraint (green line) had the highest effect on PDO dispersion at the end of the culture, but only 1.7%, validating the global sensitivity analysis.

The PBM predictions were performed assuming a 30% RSD of biomass composition (precursors and macromolecules) and kinetic parameters (acetic acid production and death constants). However, there is uncertainty in the selection of these RSD values using the *Clostridium* sp IBUN 158B strain. Therefore, we evaluated three RSD values for biomass composition: 10%, 20%, and 30%. The results are shown in S4 File as follows: random profiles are in Fig C, and RSD profiles of PDO production are presented in Fig D. As shown in part (a) of Fig D in S4 File, the maximum PDO dispersion decreases from 14.6 to 10.7% when biomass RSD drops from 30% to 10%, but no differences in the PDO dispersion are predicted at the end of the culture. Due to the previously mentioned uncertainty, we selected dispersion in biomass molecular weight as an indirect indicator of biomass RSD selection. This dispersion decreased from 3.1 to 1.0% at the range evaluated, as shown in part (b) of Fig D in S4 File. Therefore, we maintained a 30% RSD in biomass composition, because its dispersion in the biomass molecular weight (3.1%) was similar to the experimental values reported in other studies (2.75% and 2.51%, respectively, both using *E*. *coli* cultures) [71, 92]. The RSD of the kinetic parameters were also maintained at 30%, supported mainly by Mönier *et*. *al*. [93], who reported RSDs ranging from 28.4 to 33.3%.

**Fig 7:**
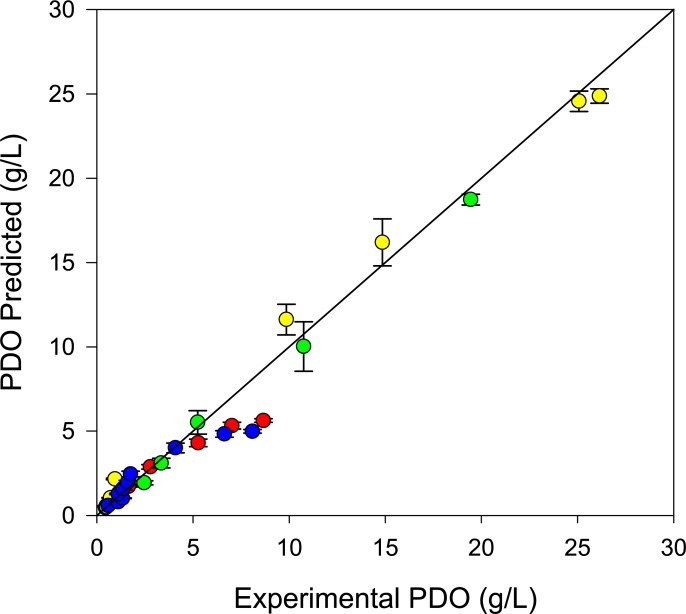
Comparison of experimental and predicted PDO values by the dynamic population balance model (PBM). Error bars correspond to predicted standard deviation obtained by PBM. Notation: culture 1 at glycerol limitation (blue dots), culture 2 at glycerol limitation (red dots), culture 1 at glycerol excess (green dots), and culture 2 at glycerol excess (yellow dots).

Based on the above results, the experimental and predicted values of PDO produced in different cultures are compared in Fig 7; the error bars are the standard deviation from PBM predictions (See Fig E in S4 File, for complete predicted profiles). Most experimental values were adequately predicted by the dynamic model. The PDO values with the lowest predictions correspond to data from the decelerated growth phase in glycerol limitation cultures, as previously mentioned. Despite these underestimated values, the linear correlation between experimental and predicted values had standard error and correlation coefficients of 4.4% and 97.6%, respectively. Consequently, using the dynamic model developed through DFBA, the previously proposed objective functions [6] and complemented using PBM, we properly predicted the PDO production of *Clostridium* sp IBUN 158B strain.

### Perturbation in culture conditions

**Fig 8:**
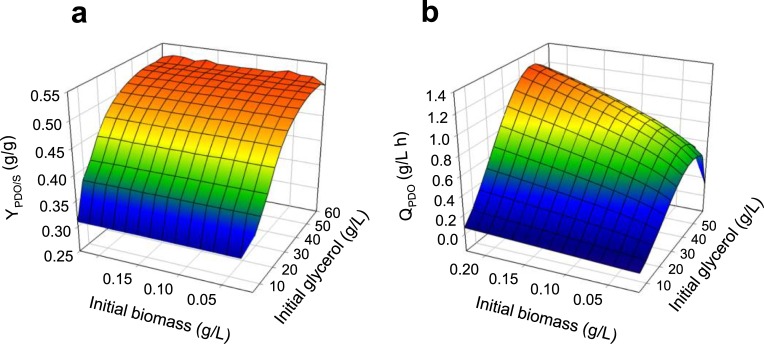
Response surfaces predicted by DFBA, varying initial concentration of biomass and glycerol in batch culture. **(a).** Predicted glycerol conversion to PDO yield (Y_PDO/S_). **(b)** Predicted PDO productivity (Q_PDO_).

Later, we evaluated different strategies to predict an increase in PDO production through perturbation in culture conditions. First, we perturbed the initial concentrations of glycerol and biomass in batch cultures; the results of conversion (Y_PDO/S_) and productivity (Q_PDO_) are shown in Fig 8. Simulations predict Y_PDO/S_ reduction at low glycerol concentrations, however at these initial glycerol levels (<5g/L) predictions cannot be trusted according to the aforementioned about kinetic adjustment error of glycerol uptake flux at these concentrations. Optimum productivity values are predicted at an initial glycerol concentration of 46 g/L, which is within the range previously reported for this strain (among 40 and 50g/L) [79, 94]. Fig 8B also shows a monotonic trend of PDO productivity in the function of initial biomass. However, according to previous studies of *Clostridium* sp. IBUN 158B, biomass inoculum has to be at the exponential phase [79, 94], meaning the maximum initial biomass concentration could be 0.12 g/L. Therefore, for batch cultures, using 0.12g/L and 46g/L as initial concentrations of biomass and glycerol, respectively, the optimum productivity predicted was 1 g/L·h, while the respective PDO final concentration was 23.5 g/L.

**Fig 9:**
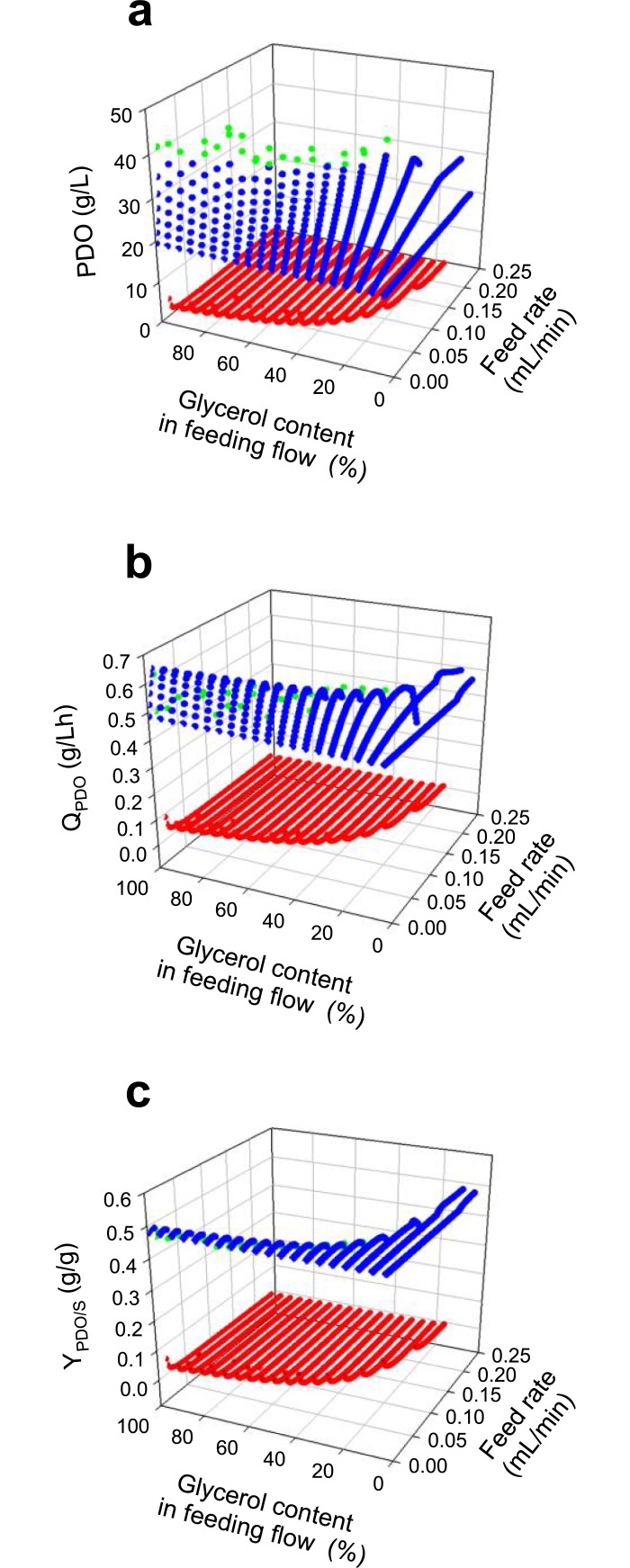
DFBA predictions of fed-batch cultures assuming constant feeding flow. **(a)** Final predicted PDO concentration. **(b)** Predicted PDO productivity (Q_PDO_). **(c)** Predicted glycerol conversion to PDO yield (Y_PDO/S_). Notation: infeasible cultures (red dots), suboptimal cultures (blue dots), and optimal cultures (green dots).

The second strategy was predicting fed-batch cultures varying the mode and the glycerol content in the feeding flow. The first feeding mode evaluated was feeding with a constant flow during the culture. Predicted results of production, productivity (Q_PDO_), and conversion yield (Y_PDO/S_) are shown in Fig 9, where glycerol concentration in feeding flow was also evaluated (See Fig A in S5 File for front, side and top views of Fig 9). An inhibitory zone is observed, caused either by an over feeding flow or an overload of glycerol in the feeding flow, in other words it is the infeasible zone with no PDO production and is presented with red dots. The zone with no inhibition but with suboptimal production is represented with blue dots, while the Pareto frontier of PDO production is shown with green dots. Excluding the infeasible zone, Y_PDO/S_ yield values remained constant, while the maximum production of PDO was 46.9 g/L, and productivity Q_PDO_ was up to 0.64 g/L·h. For the same initial conditions, fed-batch cultures enhanced production and productivity near to 144% and 28%, respectively, compared to batch cultures. However, this is still lower than experimental results using other *Clostridium* strains, wherein some production and productivity values were 70.8 g/L and 0.71 g/L·h [87] or 61.2 g/L and 1.02 g/L·h [95], respectively.

**Fig 10:**
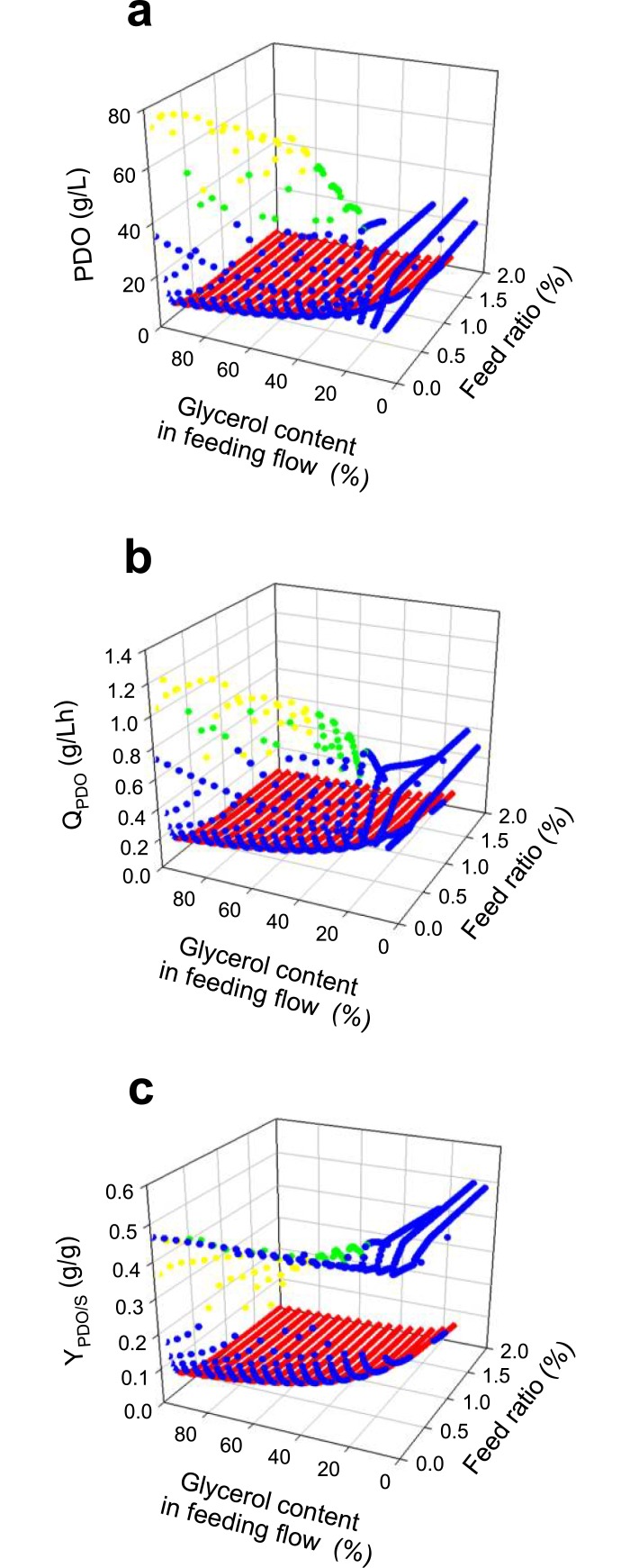
DFBA predictions of fed-batch cultures assuming feeding flow coupled to pH control. **(a)** Final predicted PDO concentration. **(b)** Predicted PDO productivity (Q_PDO_). **(c)** Predicted glycerol conversion to PDO yield (Y_PDO/S_). Notation: infeasible cultures (red dots), suboptimal cultures (blue dots), cultures with unconsumed glycerol (yellow dots), and optimal cultures (green dots).

**Fig 11:**
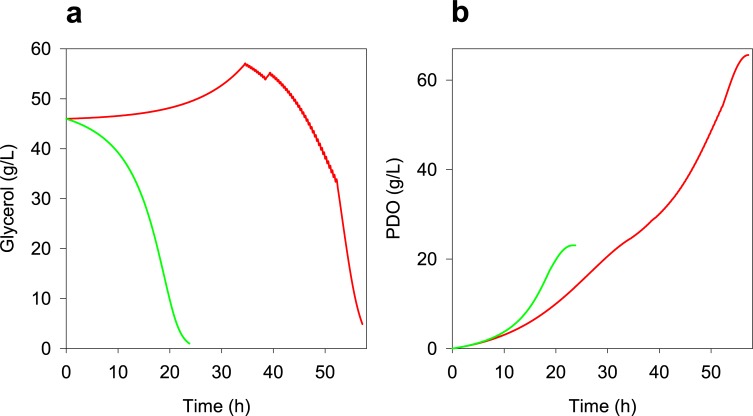
Comparison of optimum profiles predicted using DFBA. **(a)** Glycerol consumption predicted. **(b)** Predicted PDO formation. Notation: optimum prediction of batch culture (green lines) and optimum prediction of fed-batch culture (red lines).

Given the above and due to the results of Reimann *et*. *al*. [96], who improved PDO production from 47.5 to 70.3 g/L using a feeding flow coupled to the pH control (*i*.*e*. exponential feeding flow during the culture), we evaluated a final strategy where the feeding flow was proportional to proton (H^+^) production. According to Fig 10, the new feeding mode also presents an infeasible zone (red dots) and a suboptimal zone (blue dots) (See Fig B in S5 File for front, side and top views of Fig 10). Fig 10 also shows that lower Y_PDO/S_ yields are predicted compared with the constant feeding flow, which is caused by higher PDO productions and inhibiting growth by product. Therefore, the better cultures (green dots) were selected using a minimum mass conversion of 45% and a maximum concentration of glycerol at the end of the culture of 5g/L as constraints, avoiding scenarios with unconsumed glycerol (yellow dots). Therefore, the best scenario predicted a final PDO production of 66.1 g/L with a productivity of 1.15 g/L·h, which is shown in Fig 11 along with the optimum batch culture predicted. Fed-batch results are within the expected range according to the experimental values of different *Clostridium* strains [4, 5, 87, 95, 97–100], allowing to suggest that after the optimization of production and purification, this strain could be adequate to produce PDO industrially, taking advantage of glycerol co-produced during biodiesel obtainment.

## Conclusions

The *i*Cbu641 GSM model, previously validated at steady state through prediction of different *Clostridium butyricum* cultures, was employed to predict dynamic cultures. Such dynamic-state predictions were obtained using the DFBA direct approach and the objective functions proposed at steady state. We proved that the dynamic model not only predicts *Clostridium* sp IBUN 158B growth and its PDO production but also validates the objective functions proposed previously [6]. We also observed the dynamic relation between PDO production and the allosteric constraint of acetic acid secretion and its respective effect on production of other acids, such as lactic acid, which was validated after performing MPSA and PRCC sensitivity analyses. Sensitivity analyses also allow us to find that kinetic parameters of glycerol uptake flux, obtained from *Clostridium* sp IBUN 158B cultures, had the highest effect on PDO predictions, whereas the other 57 input parameters evaluated, obtained from other *Clostridium* cultures, had the lowest effect, which were used later in a PBM. Regarding this PBM developed, we quantified the heterogeneity of PDO caused by cell variability, obtaining an adjustment near to 98% to predict PDO production. Furthermore, we proposed dynamic simulations of fed-batch cultures with a strategy of feeding coupled to growth, where PDO production could increase up to three times in comparison to batch cultures. Therefore, we predicted that *Clostridium* sp IBUN 158B could reach reported PDO yields of different PDO-producing *Clostridium* strains [87, 96, 97, 100–102].

We propose the dynamic model as a valid tool to predict a wide variety of scenarios that would otherwise be experimentally demanding in terms of time and resources. Future research could couple the *i*Cbu641 GSM model and DFBA predictions with downstream processes, allowing for the estimation of the technical-economic feasibility of a hypothetical industrial process [103, 104]. The model also could be used to design efficient automatic control systems that amortize unwanted oscillatory processes during cultures in bioreactors, as is the case of designing adequately an automatized control system of the feeding flow of fed-batch cultures [105–107].

## Supporting information

S1 File Comparison of DFBA predictions using different orthogonal polynomials. (PDF)

S2 File Adjustment of the kinetic models used as constraints. (PDF)

S3 File Random profiles used in the global sensitivity analyses. (PDF)

S4 File Development of the population balance model. (PDF)

S5 File Front, side and top views of Figs 9 and 10. (PDF)

## Abbreviations

DA: Direct approach
DFBA: Dynamic flux balance analysis
DOA: Dynamic optimization approach
FAST: Fourier amplitude sensitivity test
FBA: Flux balance analysis
GSM: Genome-scale metabolic
K-S: Kolmogorov–Smirnov
LFP: Linear-fractional programming
MPSA: Multi-parametric sensitivity analysis
MSE: Mean squared error
NGAM: Non-growth–associated maintenance
NLP: Nonlinear programming
ODE: Ordinary differential equation
PBM: Population balance model
PDO: 1,3-propanediol
PRCC: Partial rank correlation coefficient
RCM: Reinforced Clostridial medium
RSD: Relative standard deviation
SOA: Static optimization approach
UFLC: Ultra-fast liquid chromatography
