# Supplementary material for: Clostridium butyricum population balance model: Predicting dynamic metabolic flux distributions using an objective function related to extracellular glycerol content
Source: PLoS One. 2018 Dec 20;13(12):e0209447. doi: 10.1371/journal.pone.0209447 (PMC6301710; doi:10.1371/journal.pone.0209447)
Supplement: S1 File — (PDF) [file pone.0209447.s001.pdf]

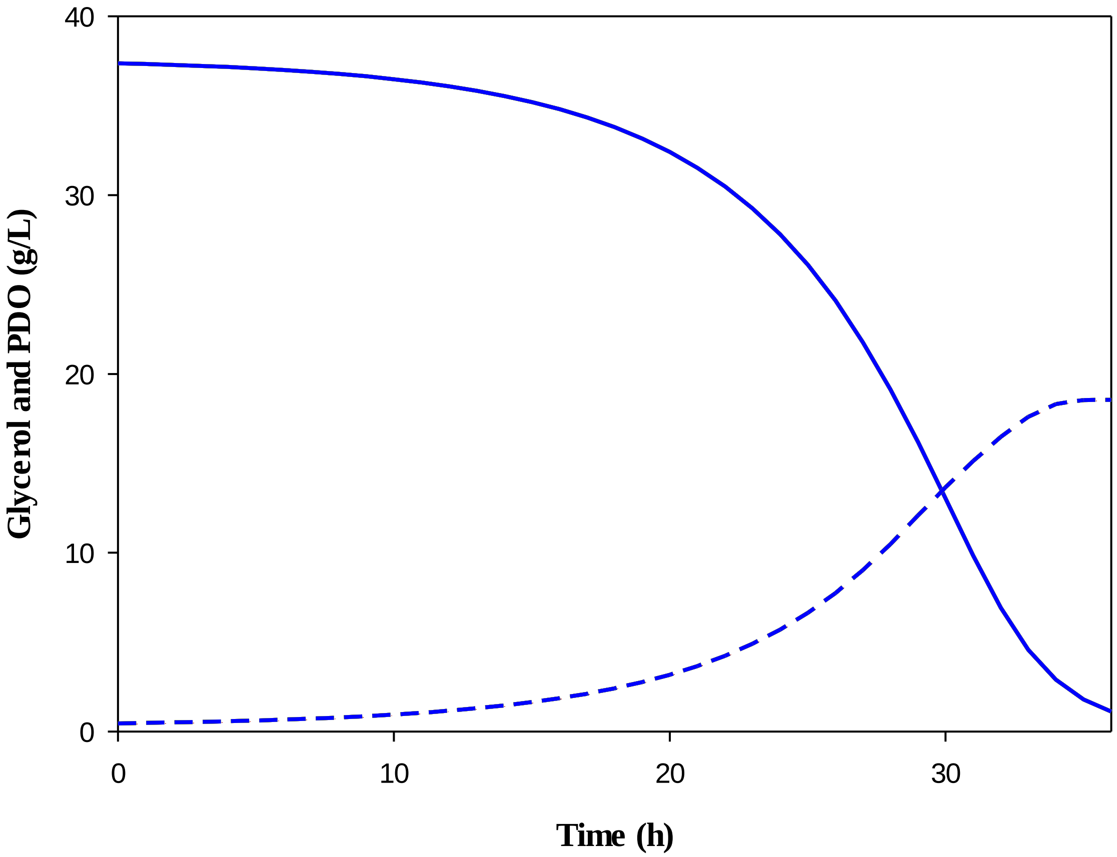

Fig A. Comparison of predicted glycerol and PDO concentrations using DFBA with different orthogonal polynomials: Notation: glycerol concentrations (continuous lines), PDO concentrations (short dashed lines). The orthogonal polynomials are: Chebyshev of the first kind (black lines), Chebyshev of the second kind (red lines), Legendre (green lines), Laguerre (yellow lines), and Hermite (blue lines).
