# Supplementary material for: Clostridium butyricum population balance model: Predicting dynamic metabolic flux distributions using an objective function related to extracellular glycerol content
Source: PLoS One. 2018 Dec 20;13(12):e0209447. doi: 10.1371/journal.pone.0209447 (PMC6301710; doi:10.1371/journal.pone.0209447)
Supplement: S2 File — (PDF) [file pone.0209447.s002.pdf]

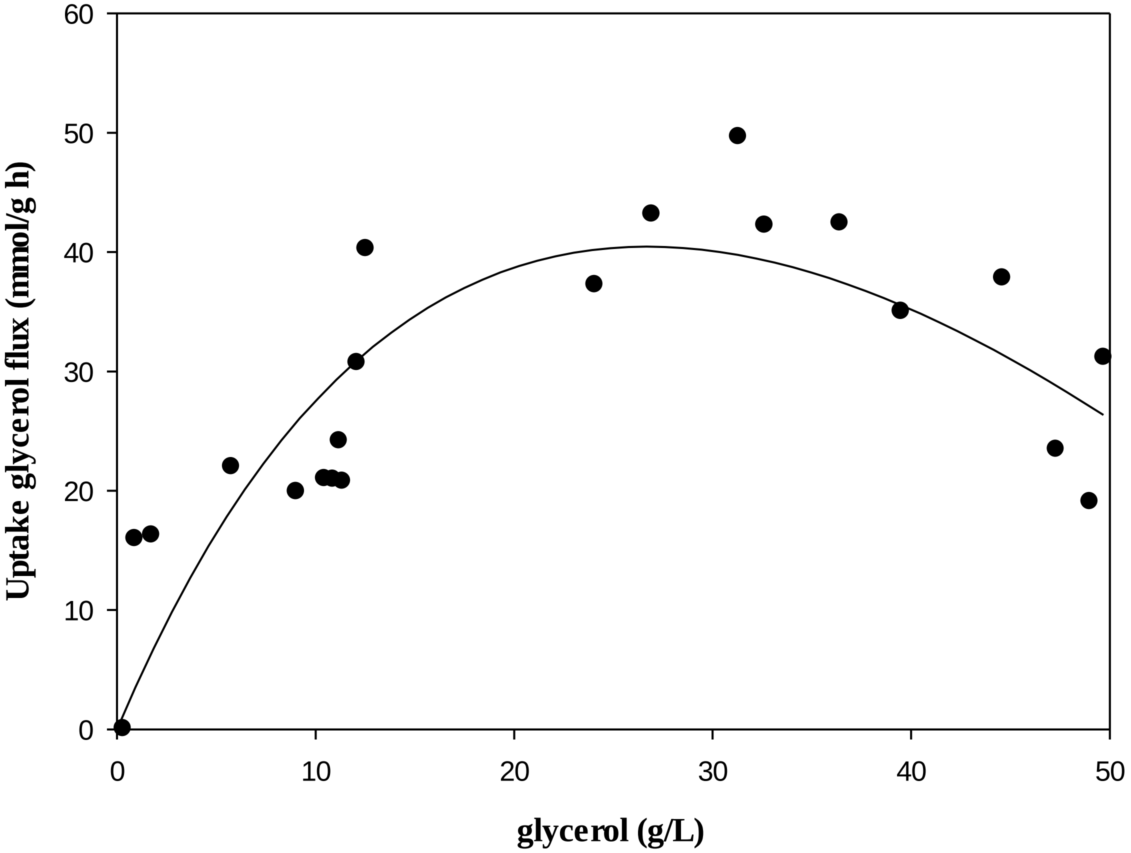

Fig A. Adjustment of the kinetic model of glycerol uptake flux in function of extracellular content of glycerol. Notation: (——) kinetic model, (●) experimental data from three *Clostridium* sp. IBUN 158B cultures: the first was one culture at glycerol limitation, the second was one culture at glycerol excess, both from this study, the third culture was performed at glycerol excess by Aragon [1].

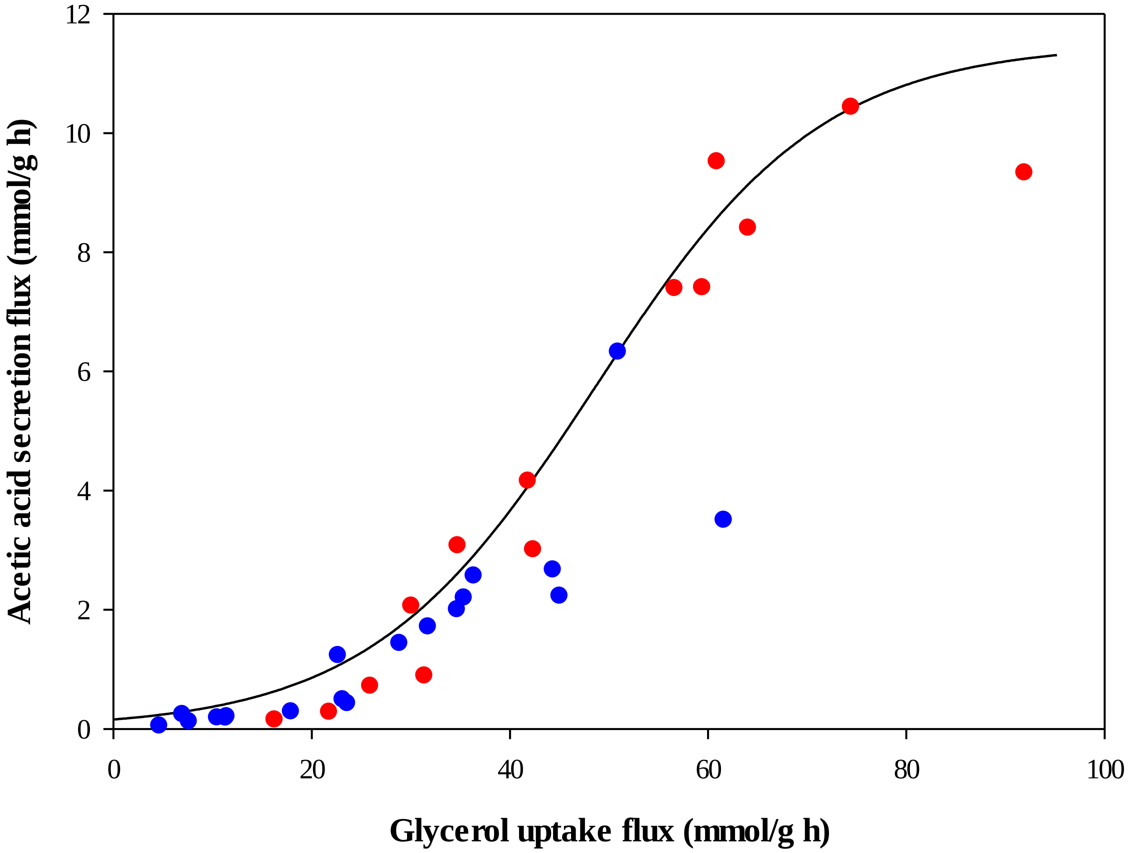

Fig B. Adjustment of the kinetic model of acetic acid secretion flux in function of glycerol uptake flux. Notation: (——) kinetic model, (●) experimental data from Solomon *et al*. [2], (●) experimental data from Papanikolaou *et al* [3].

References

1. Aragón OL. Estudio de la viabilidad técnica de la producción de 1,3 – propanodiol (1,3-pd) a partir de glicerol con nuevas cepas colombianas de Clostridium sp. a nivel laboratorio [Microbiology Thesis]: Universidad Nacional de Colombia, sede Bogotá; 2007.
2. Solomon BO, Zeng AP, Biebl H, Schlieker H, Posten C, Deckwer WD. Comparison of the energetic efficiencies of hydrogen and oxychemicals formation in Klebsiella pneumoniae and Clostridium butyricum during anaerobic growth on glycerol. Journal of Biotechnology. 1995;39(2):107-17. doi: 10.1016/0168-1656(94)00148-6. PubMed PMID: 7755965.
3. Papanikolaou S, Ruiz-Sanchez P, Pariset B, Blanchard F, Fick M. High production of 1,3-propanediol from industrial glycerol by a newly isolated Clostridium butyricum strain. Journal of Biotechnology. 2000;77(2-3):191-208. doi: 10.1016/S0168-1656(99)00217-5. PubMed PMID: 10682279.
