# Supplementary material for: Clostridium butyricum population balance model: Predicting dynamic metabolic flux distributions using an objective function related to extracellular glycerol content
Source: PLoS One. 2018 Dec 20;13(12):e0209447. doi: 10.1371/journal.pone.0209447 (PMC6301710; doi:10.1371/journal.pone.0209447)
Supplement: S3 File — (PDF) [file pone.0209447.s003.pdf]

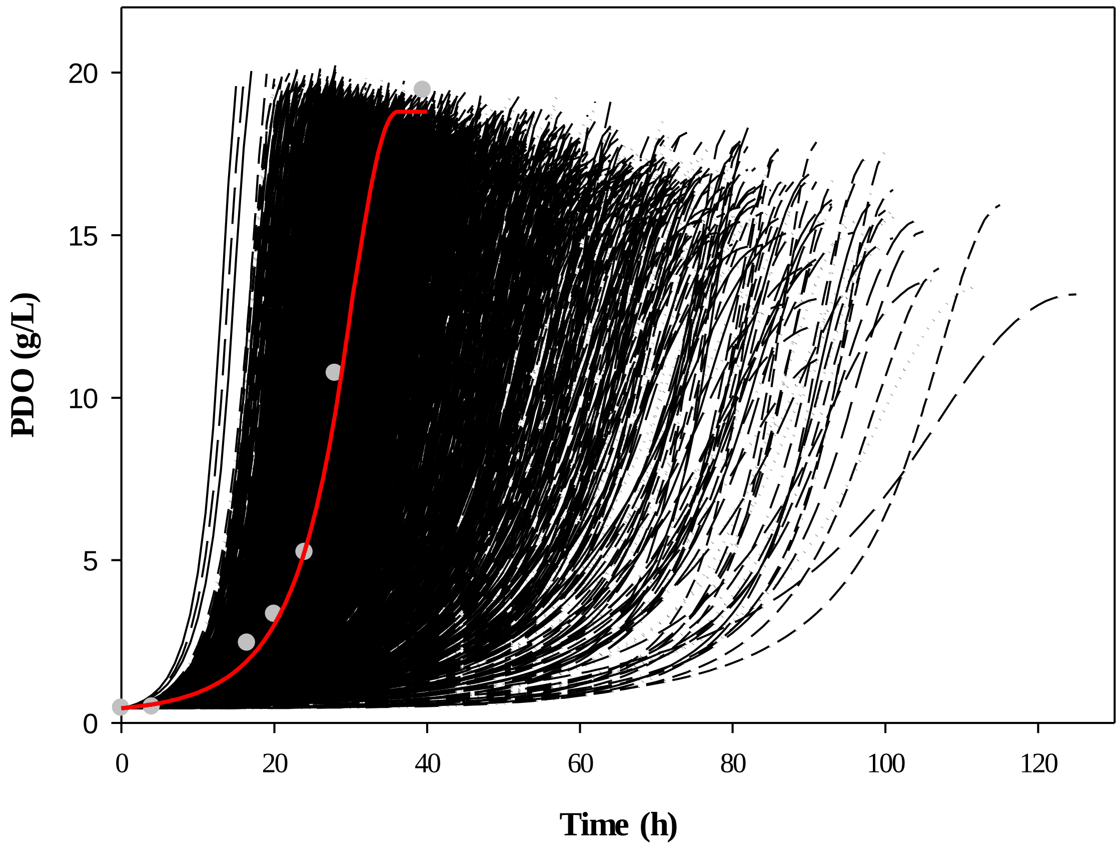

Fig A. Comparison of experimental PDO produced and 2280 profiles randomly predicted using the Monte Carlo method in DFBA. Varying the composition of 44 precursors, eight macromolecules, three kinetic parameters of acetic acid secretion flux, two kinetic parameters of cellular death, and three kinetic parameters of glycerol uptake flux. Notation: random profiles (black lines), profile at central conditions (red line), experimental values (gray dots).

.
