# Supplementary material for: Clostridium butyricum population balance model: Predicting dynamic metabolic flux distributions using an objective function related to extracellular glycerol content
Source: PLoS One. 2018 Dec 20;13(12):e0209447. doi: 10.1371/journal.pone.0209447 (PMC6301710; doi:10.1371/journal.pone.0209447)
Supplement: S4 File — (PDF) [file pone.0209447.s004.pdf]

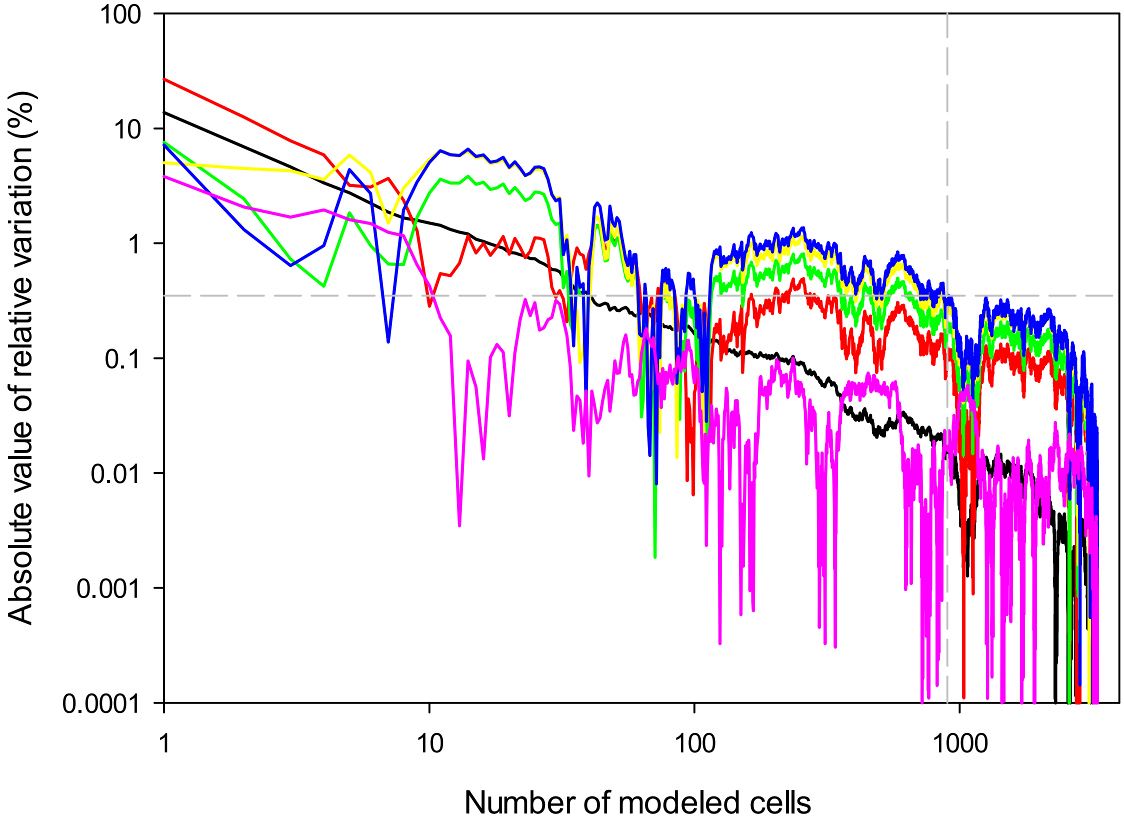

Fig A. Absolute value of relative variation between number of modelled cells and the maximum number of modelled cells (3300) randomly predicted by DFBA considering perturbation of all 57 input parameters using the Monte Carlo method. Notation: fourth hour of culture (black line), 16th hour of culture (red line), 20th hour of culture (green line), 24th hour of culture (yellow line), 28th hour of culture (blue line), 40th hour of culture (pink line), 900 modeled cells (Vertical dashed line), relative variation equal to 0.35% (horizontal dashed line)

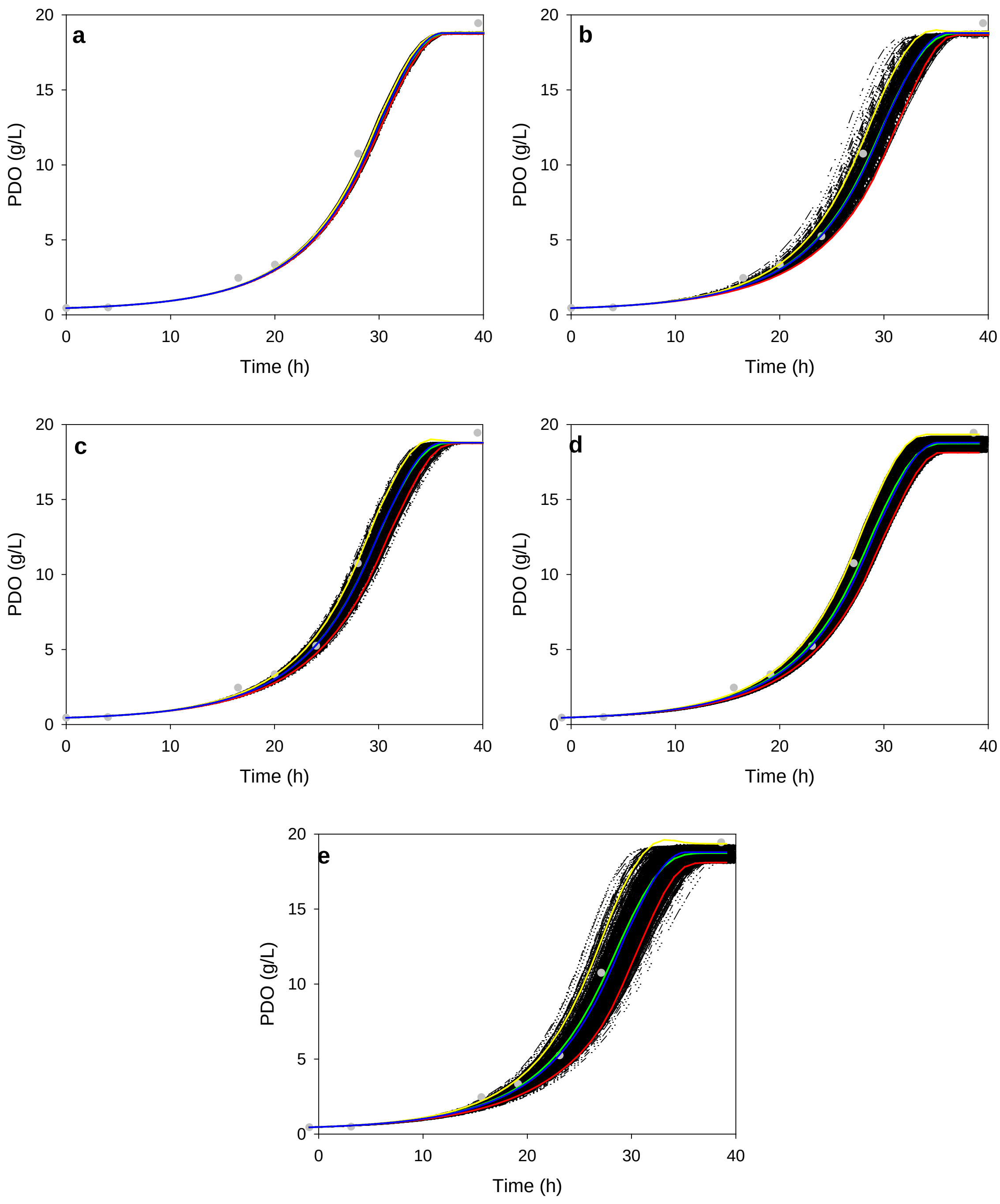

Fig B. Comparison of experimental PDO produced and profiles randomly predicted by DFBA considering perturbation in input parameters using the Monte Carlo method. Perturbation of the following: **(a)** Composition of 44 precursors. **(b)** Composition of eight macromolecules. **(c)** Two kinetic parameters of cellular death. **(d)** Three kinetic parameters of acetic acid secretion flux. **(e)** The 57 input parameters simultaneously. Notation: random profiles (black lines), profile at central conditions (blue lines), average of random profiles (green lines), upper limit with 95% confidence (yellow lines), lower limit with 95% confidence (red lines), experimental values (gray dots). RSD for all input parameters was 30%.

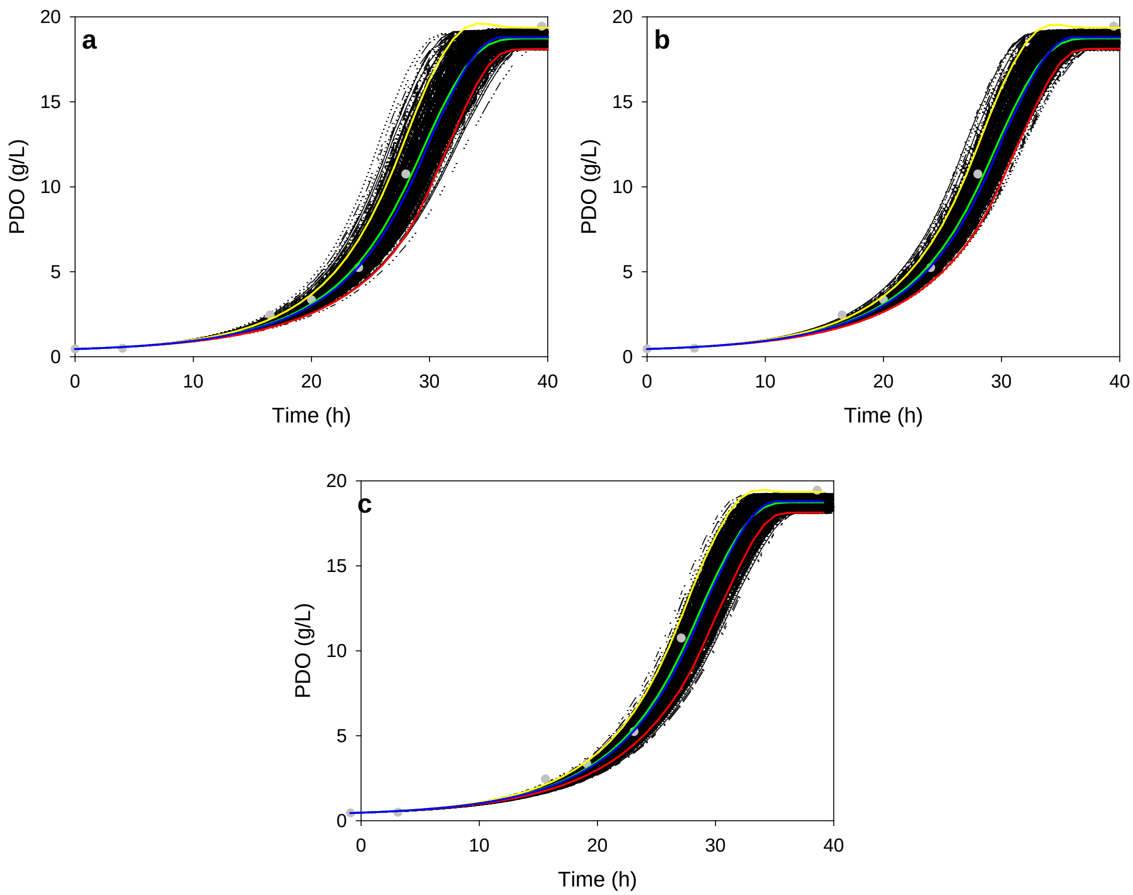

Fig C. Comparison of experimental PDO produced and profiles randomly predicted by DFBA considering perturbation in biomass composition using the Monte Carlo method. Biomass composition (precursors and macromolecules) perturbation using RSD equal to the following: **(a)** 30%, **(b)** 20%, and **(c)** 10%. Notation: random profiles (black lines), profile at central conditions (blue lines), average of random profiles (green lines), upper limit with 95% confidence (yellow lines), lower limit with 95% confidence (red lines), experimental values (gray dots). RSD for all kinetic input parameters was 30%.

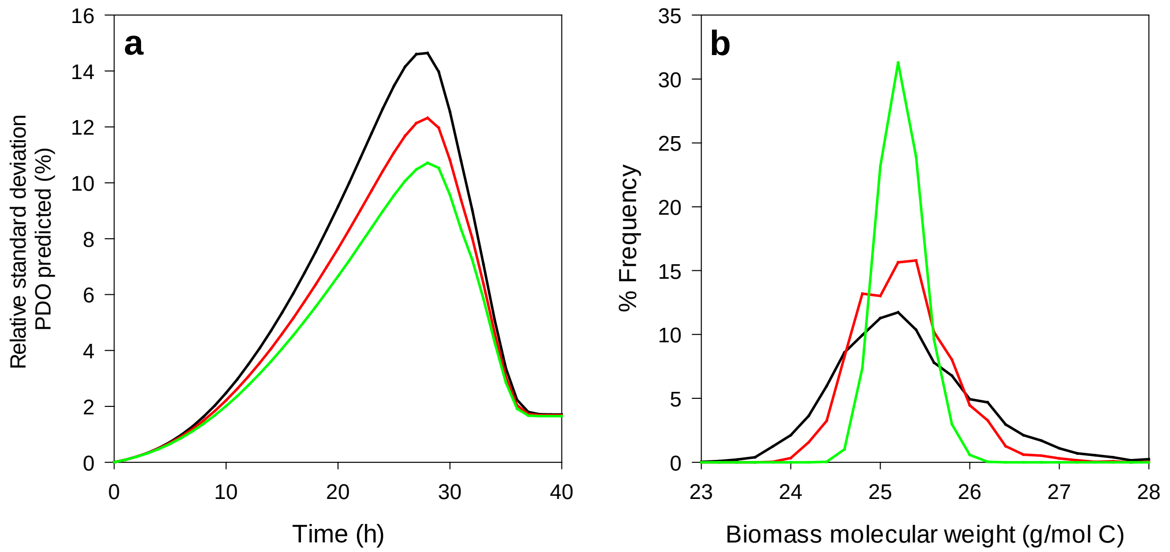

Fig D. Effect of the RSD of biomass composition (precursors and macromolecules) in a population balance model (PBM). (a) RSD of predicted PDO profiles. (b) Population distribution represented as the molecular weight of the biomass. Notation: RSDs were 30% (black lines), 20% (red lines), 10% (green lines). RSD for all kinetic input parameters was 30%.

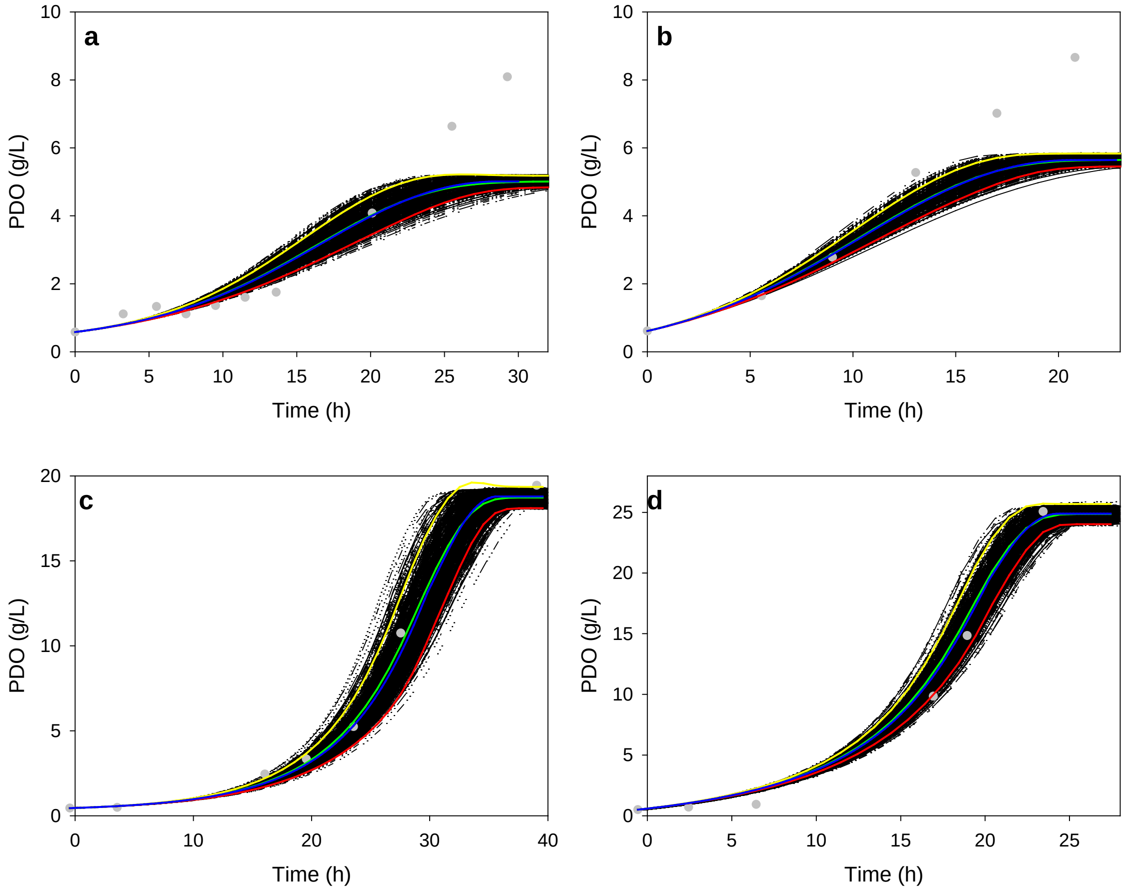

Fig E. Comparison of experimental PDO produced and profiles randomly predicted by the dynamic population balance model. **(a)** Culture 1 at glycerol limitation. **(b)** Culture 2 at glycerol limitation. **(c)** Culture 1 at glycerol excess. **(d)** Culture 2 at glycerol excess. Notation: random predictions (black), non-segregated prediction (blue), average segregated profile predictions (green), upper limit 95% confidence (yellow), interior limit 95% confidence (red). Experimental values in points. RSD was 30% for the 44 precursors, eight macromolecules, two kinetic parameters of cell death, and three kinetic parameters of the formation of acetic acid secretion flux.
