# Supplementary material for: Clostridium butyricum population balance model: Predicting dynamic metabolic flux distributions using an objective function related to extracellular glycerol content
Source: PLoS One. 2018 Dec 20;13(12):e0209447. doi: 10.1371/journal.pone.0209447 (PMC6301710; doi:10.1371/journal.pone.0209447)
Supplement: S5 File — (PDF) [file pone.0209447.s005.pdf]

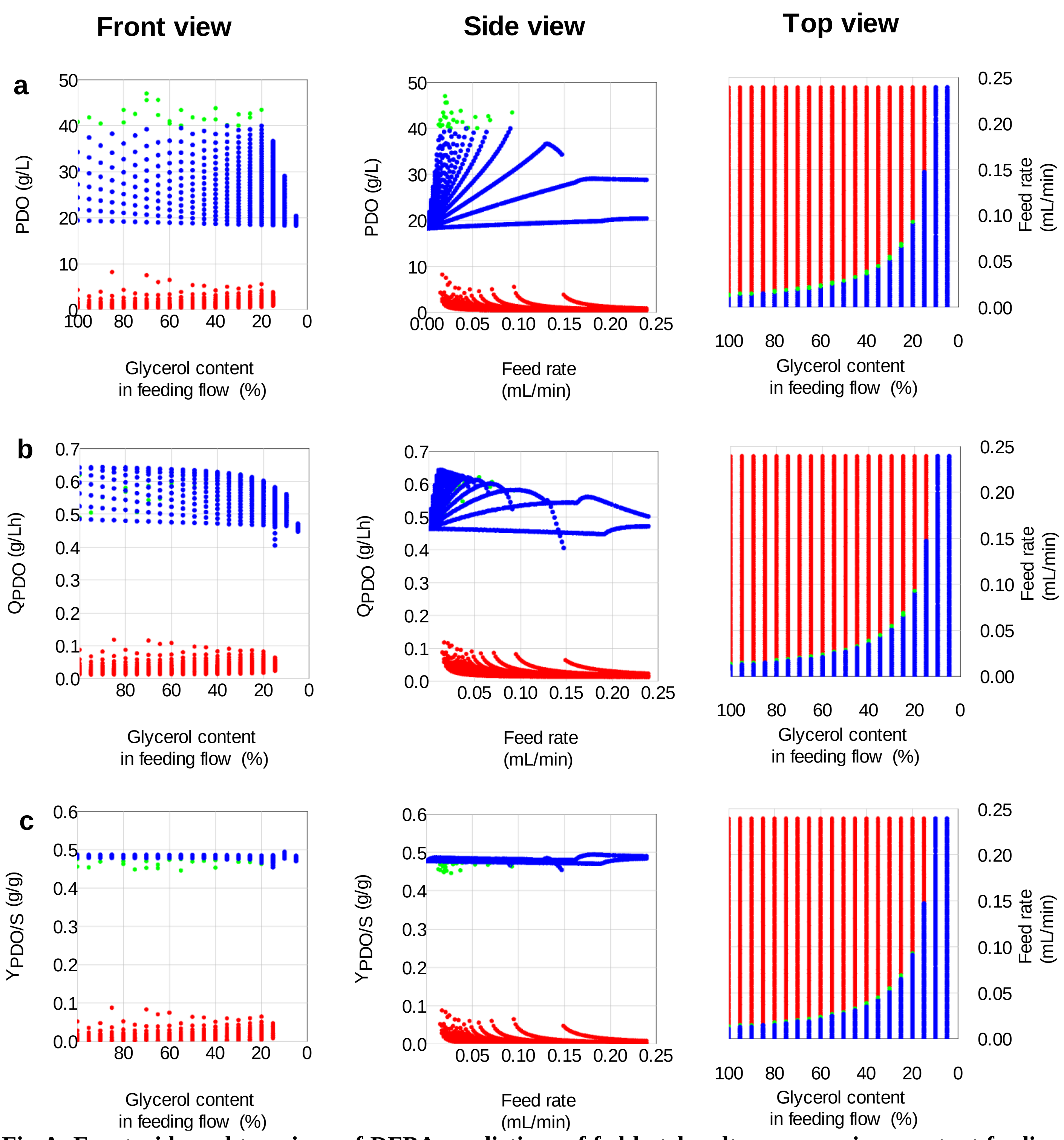

Fig A. Front, side and top views of DFBA predictions of fed-batch cultures assuming constant feeding flow. (a) Final predicted PDO concentration. **(b)** Predicted PDO productivity (Q_{PDO}). **(c)** Predicted glycerol conversion to PDO yield ($Y_{PDO/S}$). Notation: infeasible cultures (red dots), suboptimal cultures (blue dots), and optimal cultures (green dots).

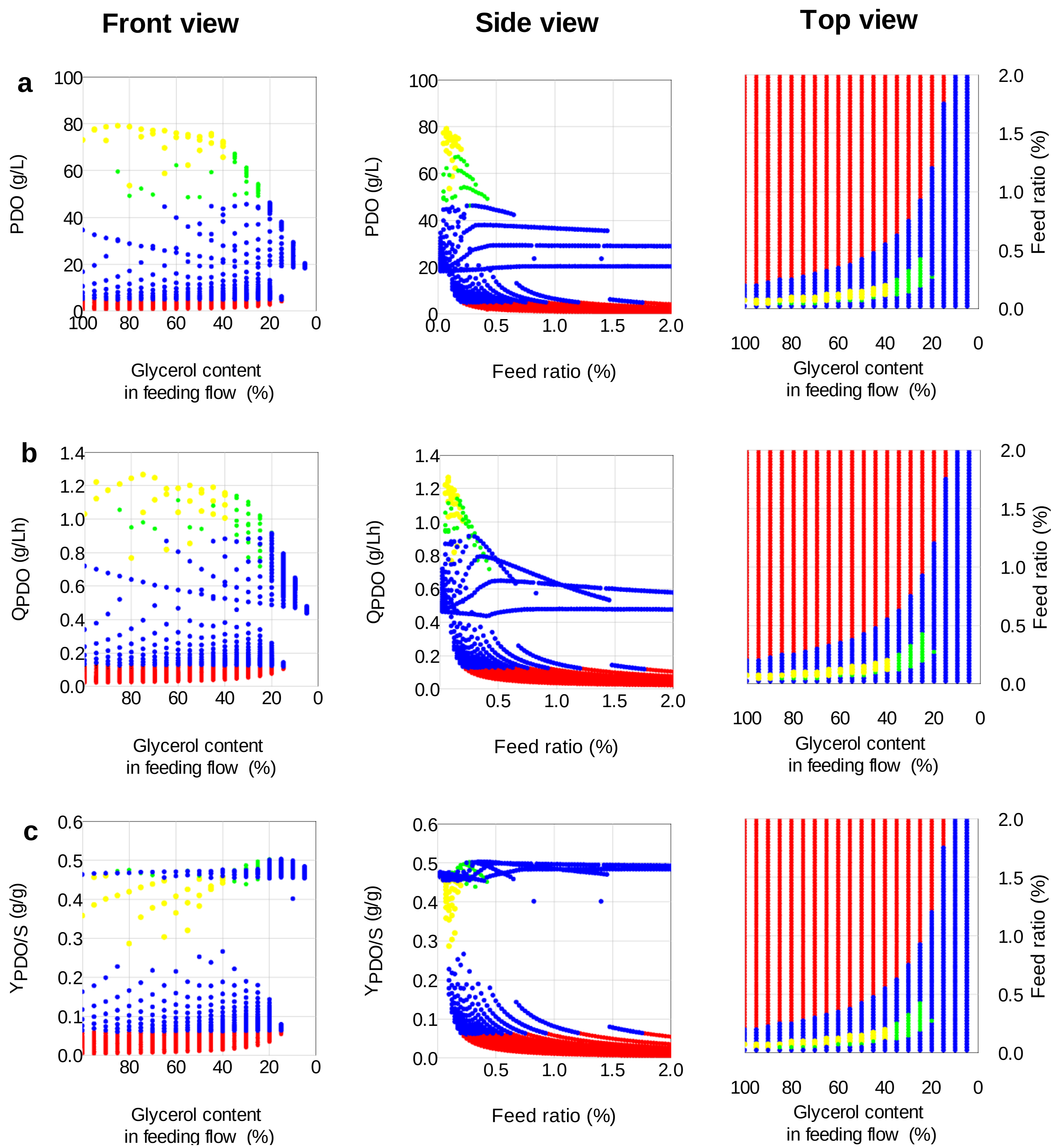

Fig B. Front, side and top views of DFBA predictions of fed-batch cultures assuming feeding flow coupled to pH control. (a) Final predicted PDO concentration. **(b)** Predicted PDO productivity (Q_{PDO}). **(c)** Predicted glycerol conversion to PDO yield ($Y_{PDO/S}$). Notation: infeasible cultures (red dots), suboptimal cultures (blue dots), cultures with unconsumed glycerol (yellow dots), and optimal cultures (green dots)
